# Circular RNA circLOC101928570 suppresses systemic lupus erythematosus progression by targeting the miR-150-5p/c-myb axis

**DOI:** 10.1186/s12967-022-03748-2

**Published:** 2022-11-26

**Authors:** Xingwang Zhao, Rui Dong, Zhongwei Tang, Juan Wang, Chunyou Wang, Zhiqiang Song, Bing Ni, Longlong Zhang, Xiaochong He, Yi You

**Affiliations:** 1 Department of Dermatology, Southwest Hospital, Army Medical University (Third Military Medical University), 30 Gaotanyan Street, Shapingba District, Chongqing, 400038 People’s Republic of China; 2 Chongqing International Institute for Immunology, 13 Tianchi Avenue, Banan District, Chongqing, 401320 People’s Republic of China; 3 Department of Pathophysiology, College of High Altitude Military Medicine, Army Medical University (Third Military Medical University), 30 Gaotanyan Street, Shapingba District, Chongqing, 400038 People’s Republic of China; 4 State Key Laboratory for Conservation and Utilization of Bio-Resources & Key Laboratory for Microbial Resources of the Ministry of Education, School of Life Science, Yunnan University, 2 Cuihu North Road, Wuhua District, Kunming, Yunnan 650091 People’s Republic of China; 5 Department of Nursing Administration, Faculty of Nursing, Army Medical University, (Third Military Medical University), 30 Gaotanyan Street, Shapingba District, Chongqing, 400038 People’s Republic of China

**Keywords:** Systemic lupus erythematosus, circLOC101928570, miR-150-5p, c-myb, Biomarker

## Abstract

**Background:**

Accumulating evidence supports the implication of circular RNAs (circRNAs) in systemic lupus erythematosus (SLE). However, little is known about the detailed mechanisms and roles of circRNAs in the pathogenesis of SLE.

**Methods:**

Quantitative real-time PCR was used to determine the levels of circLOC101928570 and miR-150-5p in peripheral blood mononuclear cells of SLE. Overexpression and knockdown experiments were conducted to assess the effects of circLOC101928570. Fluorescence in situ hybridization, RNA immunoprecipitation, luciferase reporter assays, Western blot, flow cytometry analysis and enzyme-linked immunosorbent assay were used to investigate the molecular mechanisms underlying the function of circLOC101928570.

**Results:**

The results showed that the level of circLOC101928570 was significantly downregulated in SLE and correlated with the systemic lupus erythematosus disease activity index. Functionally, circLOC101928570 acted as a miR-150-5p sponge to relieve the repressive effect on its target c-myb, which modulates the activation of immune inflammatory responses. CircLOC101928570 knockdown enhanced apoptosis. Moreover, circLOC101928570 promoted the transcriptional level of IL2RA by directly regulating the miR-150-5p/c-myb axis.

**Conclusion:**

Overall, our findings demonstrated that circLOC101928570 played a critical role in SLE. The downregulation of circLOC101928570 suppressed SLE progression through the miR-150-5p/c-myb/IL2RA axis. Our findings identified that circLOC101928570 serves as a potential biomarker for the diagnosis and therapy of SLE.

**Supplementary Information:**

The online version contains supplementary material available at 10.1186/s12967-022-03748-2.

## Introduction

Systemic lupus erythematosus (SLE) is a typical autoimmune disease characterized by the production of autoantibodies, the deposition of immune complexes and the impairment of multiorgan functions. The pathogenesis of SLE is not very clear, and previous studies have demonstrated that SLE results from the complex interaction between genetic and environmental exposures [1, 2]. The study of SLE biomarkers is critical for early diagnosis, disease activity monitoring, assessment of the likelihood and extent of organ damage and discovery of new therapeutic targets [3–6].

Circular RNAs (circRNAs) are a new type of noncoding RNA that are widely distributed in mammals. CircRNAs are single-stranded transcripts generated by back-splicing and characterized by covalently linked head-to-tail closed loop structures with neither 5’-3’ polarity nor a polyadenylated tail in eukaryotic cells [7, 8]. CircRNAs show high stability compared with linear RNAs and exhibit a cell type- or developmental stage-specific expression pattern [9]. Due to their high abundance, stability, and unique expression pattern, circRNAs are of potential utility for clinical diagnosis and prognosis. Along with the development of RNA sequencing and bioinformatics, large numbers of circRNAs have been successfully identified. Increasing evidence suggests that circRNAs are involved in the pathogenesis of a variety of diseases, including autoimmune diseases [10]. Many studies have demonstrated that circRNAs control gene expression at the transcriptional, posttranscriptional, and translational levels through distinct mechanisms, including acting as miRNA sponges, interacting with proteins or DNAs and encoding small peptides [11–14]. However, our current understanding of circRNAs in SLE is limited and needs further investigation.

In this study, we aimed to analyze circRNA profiles expressed in PBMCs of SLE patients and to investigate whether circLOC101928570 is differentially expressed and closely related to the disease activities of SLE patients. Several studies have shown that overexpression of miR-150 results in a downregulation of transcription factor c-Myb [15, 16]. Given the implication of miR-150 in systemic lupus erythematosus (SLE) and the roles of circRNAs in sponging miRNA and gene regulation, it is appealing to speculate that circLOC101928570 may associate with SLE and may be potential therapeutic targets for treatment of SLE[17, 18]. Here, we focused on the effect of circLOC101928570 competitively binding to miR-150-5P (also referred to as miR-150) [19] and regulating the expression of c-myb, which might regulate the transcription of IL2RA and eventually protect against disease progression. This study may provide a promising biomarker for the prevention, diagnosis and treatment of SLE.

## Methods

### Patient samples

Sixty-two SLE patients and healthy volunteers were recruited from the First Affiliated Hospital of Army Medical University between 2017 and 2020. The SLE patients included in the study fulfilled at least four of the 1997 revised criteria of the American College of Rheumatology (ACR) [20], and all the patients were diagnosed with SLE for the first time or without treatment with glucocorticoid or immunosuppressive agents for one month. The demographic, clinical, and laboratory characteristics of each subject were recorded, and disease activity was evaluated with the SLEDAI [21]. Information on SLE patients can be found in Table 1 and Additional file 1: Table S1. All participants were Han Chinese.

**Table 1:** Clinical features of SLE patients and demographic data of HCs

Characteristics	SLE patients(n = 62)	HCs(n = 62)
Age, years, median (IQR)	37.0(20.8–54)	36.0(21.5–53.4)
Female, n (%)	55(89)	55(89)
SLEDAI score, mean (IQR)	9.05(2.73–15)	N/A
Complement C3, median (IQR)	0.58(0.25–0.92)	N/A
Complement C4, median (IQR)	0.14(0.04–0.27)	N/A

*IQR* interquartile range

### RNA extraction and RNA quantitative real-time polymerase chain reaction

Samples were derived from PBMC, CD4 + T cells and CD8 + T cells in healthy human and SLE patients. And T cells were separately isolated by using EasySepTM Human CD4 + T cell Isolation Kit and EasySepTM Human CD8 + T cell Isolation Kit (STEMCELL, Canada). Total RNA was harvested and separated from PBMCs in samples via TRIzol reagent (Invitrogen, United States), and complementary DNA (cDNA) was synthesized sequentially. Two micrograms of total RNA was used to synthesize cDNA, a portion of which (1 µl, equal to 0.2 µg of cDNA) was used in a PCR assay. After reverse transcription with the PrimeScriptTM RT Reagent Kit (Takara, Japan), cDNA was amplified using SYBR Green Super Mix (Roche, Switzerland). Experimental results were analyzed through the 2-∆∆Ct method. The expression levels of miR-150-5p and nuclear circLOC101928570 were normalized to the expression of U6; in other cases, the expression levels of LOC101928570 and circLOC101928570 were normalized to the expression of β-actin mRNA. The sequences of the primers used for qRT–PCR in this study are shown in Additional file 2: Table S2.

### Ribonuclease R treatment

Total RNA (2 μg) of PBMCs was mixed with 3 U/μg ribonuclease R (Epicenter Technologies, United States) at 37 °C for 20 min. The stability of circLOC101928570 and linear LOC101928570 was determined. Relative expression levels were evaluated by qRT–PCR.

### *Fluorescence *in situ* hybridization*

The PBMC suspension was pipetted onto autoclaved glass slides, followed by dehydration with 70%, 80% and 100% ethanol. Then, hybridization was performed at 37 °C overnight in a dark, moist chamber using fluorescently labeled probes for circLOC101928570 and miR-150-5p. Briefly, circLOC101928570 was captured with a Cy3-labeled probe (5′-TGGCTTGAATAGATTGGGACTA ATA-3′), while miR-150-5p was captured with a digoxin-labeled probe (5′-TCTCCCAACCCTTGTACCAGTG-3′) for fluorescence in situ hybridization (FISH). MiR-150-5p was further visualized using the anti-digoxin rhodamine-conjugated antibody. After washing, the slices were sealed with parafilm containing DAPI. Specimens were analyzed using a Nikon inverted fluorescence microscope.

### Cell isolation and culture

Whole blood (10 ml) was collected in EDTA collection tubes from each subject, and human PBMCs were isolated by density-gradient centrifugation using Ficoll-Paque Plus (GE Healthcare Biosciences, United States) and cultured in RPMI 1640 medium (Gibco, United States) supplemented with 10% fetal bovine serum (Gibco, USA), 100 U/mL penicillin, and 100 U/mL streptomycin (Gibco, United States) at 37 °C with 5% CO_2_ for 24 h before transfection.

### Cell transfection

The circLOC101928570 overexpression plasmids and empty vector were constructed and designed by GeneChem (Shanghai, China). The miR-150-3p mimics, miR-150-5p mimics, miR-150-3p inhibitor, miR-150-5p inhibitor, miRNA mimics NC, and miRNA inhibitor NC were chemically synthesized by Riobio (Guangzhou, China). The pCDH-CMV-MCSEF1- GFP + Puro (CD513B-1) vector was used as a negative control plasmid, and the pCDH-MYB plasmid was fragmented and inserted into the pCDH-CMV-MCSEF1-GFP + Puro (CD513B-1) vector with BamHI and NotI restriction sites. The vector construction results were verified by direct sequencing. The sequence used was 5 ‘-CCGGAATTCCGGGAAAGCGTCACTTGGGGAAAA-3’. PLKO.1-puro (Addgene plasmid # 8453) was used to design short hairpin RNA, and the cells were transfected with Lipofectamine 3000 (Invitrogen, United States). The transfection process lasted 48 h.

### Luciferase reporter assay

293 T cells in 24-well plates were cotransfected with miR-150-3p/5p mimic, inhibitor, and negative control, and luciferase reporter vectors (SV40-Luc-MCS) with wild-type or mutant circLOC101928570 were designed and constructed by GeneChem (Shanghai, China). For circLOC101928570 and miR-150-3p/5p luciferase reporter assays, the circLOC101928570 sequences containing wild-type miR-150-3p/5p predicted binding sites were inserted into the region directly downstream of a cytomegalovirus (CMV) promoter-driven firefly luciferase cassette in a pCDNA3.1 vector. The IL2RA 3’ UTR sequences containing two predicted wild-type c-myb binding sites were inserted into the region directly downstream of a T7 promoter-driven firefly luciferase cassette in a psiCHECKTM-2 vector (Promega, United States). All constructs were verified by sequencing. 293 T cells were seeded into 24-well plates and cotransfected with a mixture of 1 μg of luciferase reporter plasmid and PCDH and plasmids pCDH-MYB, shNC, and shMYB. The sequence of shRNA-MYB#1 and shRNA-MYB#2 used were 5ʹ-CCGGGCGGCTGAATAGGTTGCTTGTTTCAAGAGAACAAGCAACCTATTCAGCCGCTTTTTTGGTACC-3ʹand 5'-CCGGGCACACGACAGAGATCTTTCCTTCAAGAGAGGAAAGATCTCTGTCGTGTGCTTTTTTGGTACC-3', respectivley. After 48 h, luciferase activity was measured using the Dual-Luciferase® Reporter Assay System (Promega, United States). The relative firefly luciferase activity was normalized to Renilla luciferase activity. All experiments were performed in triplicate.

### Short hairpin RNA

To stably knock down circLOC101928570 expression, Jurkat cells were cultured and infected with lentivirus carrying shRNA targeting circLOC101928570 and a negative control vector. PLKO.1-puro was used to design short hairpin RNA, and the restriction sites were AgeI (R3552S) and EcoRI (R3101T). After 1300 bp, a single restriction site, KpnI (R0142M), was used. For lentivirus packaging, HEK-293 T cells were transfected with the core plasmid PLKO.1-shRNA, the psPAX2 packaging plasmid and the pMD2.G envelope plasmid for 48 h to obtain the lentivirus supernatant. The shRNA sequences used are shown in Additional file 3: Table S3. All constructs were verified by sequence analysis. No homology to any other human genes was detected.

### Apoptosis

Double staining of Annexin V and 7-aminoactinomycin-D (7-AAD) was carried out using a PE Annexin V Apoptosis Detection Kit (BD Pharmingen™, United States) and an APC Annexin V Kit (BD Pharmingen™, United States) according to the manufacturer’s recommendations. Briefly, cells were washed with cold phosphate-buffered saline and then resuspended in binding buffer at a concentration of 1 × 10^6^ cells/ml. Then, 100 μl of solution (1 × 10^5^ cells) was transferred to a tube, and 5 μl of PE Annexin V and 5 μl of 7-AAD were added. After incubation for 15 min at room temperature in the dark and the addition of 400 μl of binding buffer, flow cytometric analysis was performed (FACScan, BD Biosciences, San Jose, CA, United States) within 1 h, and the data were analyzed with FlowJo software (Treestar, Ashland, OR). PE Annexin V ( +) or APC Annexin V ( +) and 7-AAD (-) cells represent the early stage of apoptosis, whereas PE Annexin V ( +) or APC Annexin V ( +) and 7-AAD ( +) cells are in the end stage of apoptosis or are already dead.

### Enzyme-linked immunosorbent assay (ELISA)

Concentrations of IL-4 and IFN-γ in peripheral blood serum supernatants were analyzed using a Human IL-4 Instant ELISA Kit (eBioScience, United States) and a Human IFN gamma Platinum ELISA Kit (eBioScience, United States) following the manufacturer’s instructions, respectively. The concentrations were calculated according to their corresponding standard curves.

### Prediction of ceRNAs for circLOC101928570

A mutually targeted method was applied to predict putative ceRNAs for circRNAs. To predict ceRNAs for circLOC101928570, we used circMir1.0 software to identify circLOC101928570-targeting miRNAs.

### Pull-down assay

The biotinylated circLOC101928570 probe was specifically designed and synthesized for binding to the junction site of circLOC101928570. The biotin-coupled RNA complex was pulled down by incubating the cell lysates with PierceTM Streptavidin Magnetic Beads (Thermo Fisher Scientific, United States) following the manufacturer’s instructions. The enrichment of miRNAs in the capture fractions was evaluated by qRT–PCR analysis. After reverse transcription with a Mir-X™ miRNA First Strand Synthesis Kit (Takara, Japan), complementary DNA (cDNA) was amplified using a Mir-X™ miRNA qRT–PCR TB Green® Kit (Takara, Japan), the expression of miRNAs was normalized to the expression of U6, and the probe sequences used are listed in Additional file 2: Table S2.

### RNA-binding protein immunoprecipitation (RIP)

A RIP assay was performed using a Magna RIP RNA Binding Protein Immunoprecipitation Kit (Millipore, United States) according to the manufacturer’s instructions. Briefly, PBMCs were harvested and lysed in RIP lysis buffer on ice for 30 min. After centrifugation, the supernatant was incubated with 30 μl of Protein G agarose beads (Roche, United States) and 8 μl of AGO2 (ab57113, Abcam, Cambridge, MA) antibody. After overnight incubation, the immune complexes were centrifuged and then washed six times with washing buffer. The bead-bound proteins were further analyzed using Western blotting. The immunoprecipitated RNA was subjected to qRT–PCR analysis.

### Western blot analysis

The complete proteome from PBMCs was extracted after lysis in RIPA lysis buffer (Beyotime, China) supplemented with protease inhibitors (Sigma–Aldrich, United States) and then separated via sodium dodecyl sulfate–polyacrylamide gel electrophoresis. The samples were electrotransferred to polyvinylidene fluoride membranes (Millipore, United States). After blocking with 5% fat-free milk, the membranes were treated with prepared primary antibodies: anti-c-myb (Abcam, England), rabbit IL2Rα antibody (CST, United States), and rabbit GAPDH antibody (CST, United States) were used as controls. Membranes were washed and then treated with an anti-rabbit secondary antibody (CST, United States). The blot signal was examined using Pierce ECL Western blotting Substrate (Thermo Fisher Scientific, United States) with a ChemiDoc™ Touch Imaging System (Bio–Rad, United States). The integrated density values were calculated using Quantity One software (Bio–Rad, United States).

### Flow cytometry analysis

Flow cytometry was performed on freshly isolated PBMCs, as a previous study showed that CD25 expression is affected by freezing–thaw procedures. PBMCs were stained with fluorochrome-conjugated antibodies to identify blood CD4^+^ and CD8^+^ T cell subsets by flow cytometry. The staining procedure was conducted blinded to genotype and was performed simultaneously for each pair. Prior to staining, FcR Blocking Reagent (Miltenyi Biotec, Bergisch Gladbach, Germany) was added to the PBMCs to prevent nonspecific binding. We used monoclonal antibodies specific for CD3, CD8, CD25, IFN-γ, IL-4, FOXP3 and IL17A for flow cytometry. All antibodies were purchased from BioLegend (San Diego, CA, United States). After the samples were stained for surface markers, the cells were further fixed and permeabilized, followed by staining for intracellular indicators. The stained PBMCs were washed 3 times in cold PBS/2% FCS/0.1% NaN_3_, and data were acquired on a FACS Canto II 8 color flow cytometer (BD Biosciences, San Jose, CA, United States) aiming for 30,000 acquisitions.

### Statistical analysis

Data were analyzed and visualized with GraphPad Prism 8.0 (GraphPad Software, La Jolla, CA, United States). Receiver operating characteristic (ROC) analysis was performed to differentiate patients from healthy controls, areas under the curve (AUCs), optimal threshold values, sensitivity, and specificity were calculated. Quantitative values are expressed as the means ± standard deviation (SD) of at least three independent repetitions. Statistical differences among groups were tested with an unpaired two-tailed Student’s t test. Spearman’s analysis was used to test correlation. A P value less than 0.05 was chosen to indicate statistical significance.

## Results

### Identification and characterization of circLOC101928570, a circRNA specifically downregulated in SLE

**Fig. 1:**
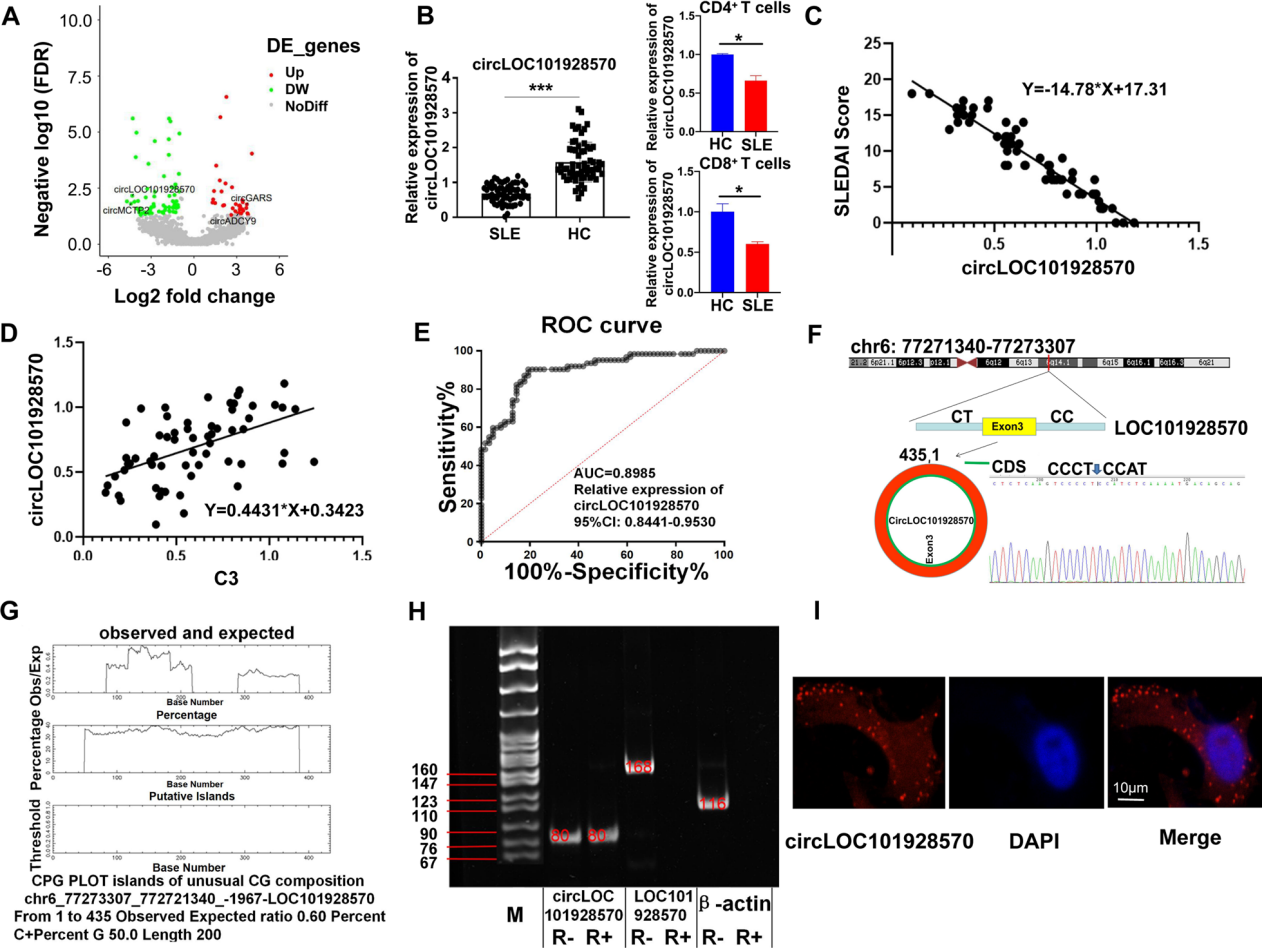
Identification and characterization of circLOC101928570, a circRNA that is significantly downregulated in SLE. **A** Volcano plot describing the profile of differentially expressed circRNAs between the control and SLE groups. The selected 4 significantly changed circRNAs are marked with green and red dots (FDR < 0.05, |fold change|> 2.0). **B** CircLOC101928570 expression was significantly lower in SLE patients. The expression of circLOC101928570 in CD4 + T cells and CD8 + T cells from HC and SLE patients. The results are represented as the mean ± SD (n = 3), *P < 0.05, ***P < 0.001. **C**, **D** Liner regression analysis for the expression of circLOC101928570 and the SLEDAI score and complement C3 level, respectively. **E** ROC curve of relative circLOC101928570 expression for differentiating the 62 patients with SLE from the 62 healthy controls. **F** Structure of the circLOC101928570 genome and transcript. CircLOC101928570 is produced by exon 3, and the junction point of circLOC101928570 was identified by qRT–PCR followed by Sanger sequencing. **G** Two CPG islands existed in the transcriptional core region of circLOC101928570. **H** Relative expression of circLOC101928570, linear LOC101928570 and actin were tested by PCR upon RNase R treatment. **I** FISH showed circLOC101928570 localization. circLOC101928570 probes were labeled with Cy3, and nuclei were stained with DAPI; scale bar: 10 µm

To identify circRNAs that contribute to SLE pathogenesis, we analyzed the circRNA RNA-seq data [22] to identify circRNAs with significant differences (*P* < 0.01) between SLE patients and healthy controls (Fig. 1A and Additional file 4: Table S4). We selected and identified 2 upregulated circRNAs and 2 downregulated circRNAs (|fold change|> 2, *P* < 0.01) that were significantly related to SLE (Additional file 4: Table S4. labeled in red), among which circLOC101928570 showed a lower level of expression in SLE patients. The circLOC101928570 was derived from the LOC101928570 gene locus and was predicted to target miR-150, a well-known SLE-associated miRNA [23, 24] and experimentally confirmed that their expression was consistent with the circRNA RNA-seq data (data not shown).

To further investigate the effects of circLOC101928570 on SLE, we verified that it was markedly downregulated in PBMCs of 62 SLE patients versus 62 healthy controls, we also checked the expression of circLOC101928570 in CD4 + and CD8 + T cells isolated by magnetic activated cell sorting (MACS) from the PBMCs of SLE patients and healthy controls. Results showed that circLOC101928570 was downregulated in the CD4 + and CD8 + T cells from SLE patients compared with the healthy controls (Fig. 1B). Liner regression analysis demonstrated that patients with lower expression of circLOC101928570 had a significantly higher SLEDAI score (Fig. 1C) and a lower complement C3 level (Fig. 1D). To assess the diagnostic value of circLOC101928570 for SLE, receiver operating characteristic (ROC) curve analysis was performed to determine the relative circLOC101928570 expression between the 62 patients and 62 healthy controls (Fig. 1E). The area under the curve (AUC) was 0.8985, and the 95% confidence interval (95% CI) was 0.8441 − 0.9530. We next evaluated the circular structure of circLOC101928570, which originated from exon 3 of the LOC101928570 gene (chr6: 77271340–77273307). Sanger sequencing validated the back-spliced junction of circLOC101928570 (Fig. 1F). Software verified that 2 CPG islands existed in the transcriptional core region of circLOC101928570 (http://www.ebi.ac.uk/emboss/cpgplot/), as shown in Fig. 1G. Then, we used the exonuclease RNase R to examine the stability of circLOC101928570. circLOC101928570 showed strong resistance to digestion by RNase R, whereas the linear RNAs of LOC101928570 and β-Actin were highly degraded (Fig. 1H). FISH results showed that circLOC101928570 was predominantly localized in the cytoplasm (Fig. 1I). Together, these data suggest that circLOC101928570 was significantly expressed at low levels in SLE patients and correlated with SLE.

### CircLOC101928570 acted as a sponge for miR-150-5p

**Fig. 2:**
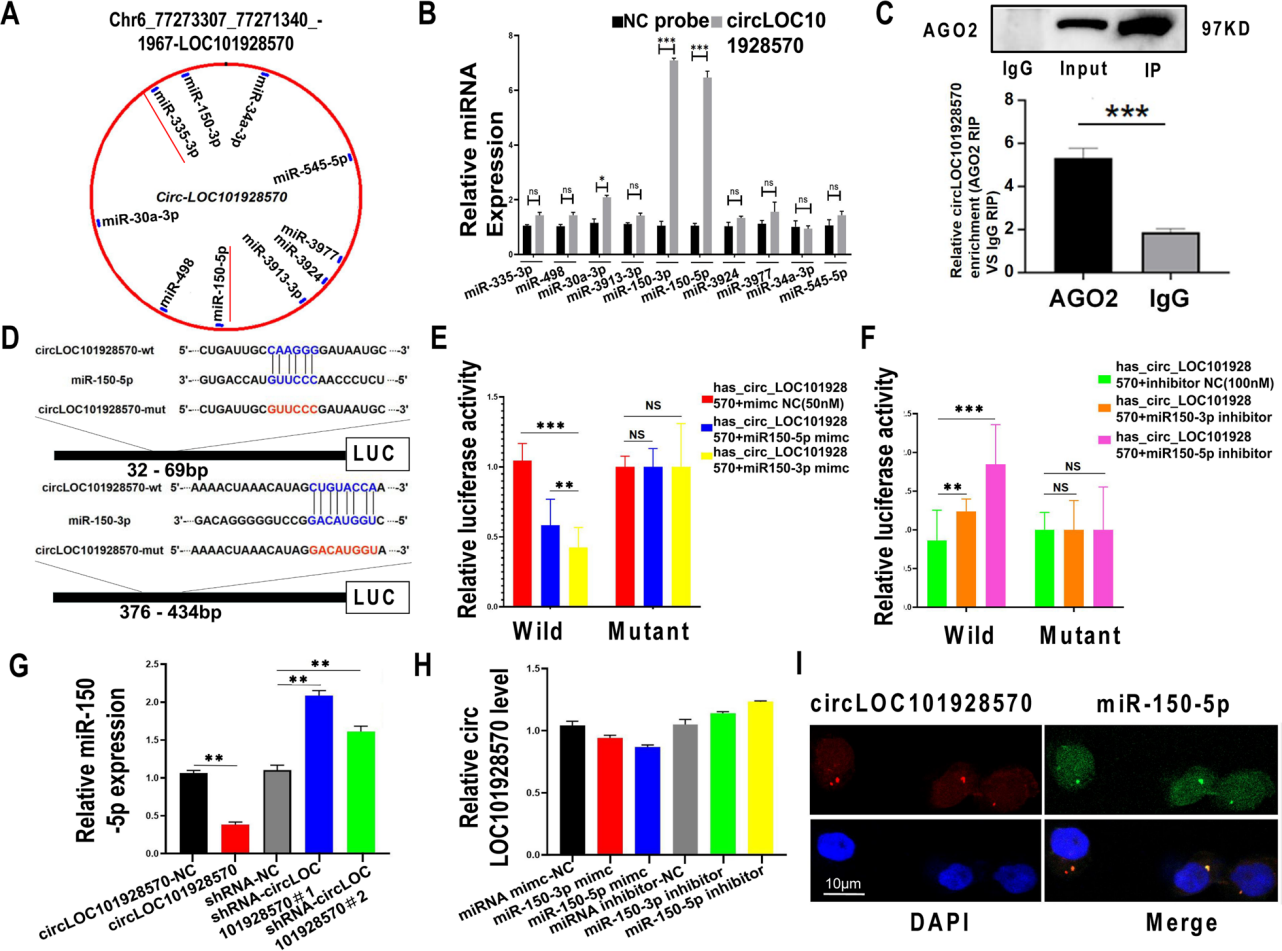
CircLOC101928570 acted as a sponge for miR-150-5p.** A** ceRNAs that could competitively bind to this exonic circRNA. MiR-150 has two targets marked with red lines. **B** The relative levels of 10 miRNA candidates in the PBMC lysates were detected by real-time PCR. miR-150-3p/5p was pulled down by circLOC101928570 in PBMCs. **C** The interaction of circLOC101928570 with miR-150 was tested by RNA immunoprecipitation (RIP) of AGO2 from HEK293T cells. Relative circLOC101928570 enrichment levels were quantified by RIP-qPCR (AGO2 RIP/IgG RIP). **D** Schematic illustration of wild-type or mutant transcripts of the circLOC101928570 luciferase reporter. **E**, **F** Luciferase reporter and effects of miR-150-3p/5p on the expression of wild-type or mutant transcripts of the circLOC101928570 luciferase reporter. Luciferase activity was normalized to the value obtained in cells transfected with NC oligonucleotides. **G** The expression of miR-150-5p was analyzed by qRT–PCR in cells transfected with circLOC101928570, mock vector, shRNA-circLOC101928570 or shRNA-NC. **H** The expression levels of circLOC101928570 were determined with qRT–PCR in cells transfected with miR-150-3p/5p mimics or inhibitor. **I** The colocalization of circLOC101928570 and miR-150-5p in PBMCs was detected using a FISH assay. CircLOC101928570 was captured with a Cy3-labeled probe (red), while miR-150-5p was captured with a digoxin-labeled probe followed by further visualization using an anti-digoxin rhodamine-conjugated antibody (green). Blue: stained with DAPI. Scale bar: 10 µm. IgG: immunoglobulin G. All data are presented as the means ± SD (n = 3). *P < 0.05, **P < 0.01, ***P < 0.001

To explore the possible mechanism of functional circLOC101928570, we identified its ceRNAs with circMir1.0 software (http://www.bioinf.com.cn/). The results showed that circLOC101928570 has two targets, i.e., the two mature products (miR-150-5p and miR-150-3p) of pre-miR-150 [25] (Fig. 2A). The miRNAs were extracted after pull-down assay, and the levels of 10 candidate miRNAs were detected by real-time PCR. MiR-150-3p/5p was abundantly pulled down by circLOC101928570 in PBMCs (Fig. 2B). To determine whether circLOC101928570 functions as a miRNA sponge, we next performed AGO2 immunoprecipitation to determine whether circLOC101928570 served as a platform for AGO2 and miR-150-5p. The results showed that circLOC101928570 binds to AGO2, and qRT–PCR results further supported this observation (Fig. 2C). To confirm that circLOC101928570 could be regulated by miR-150-3p/5p, we constructed luciferase reporters containing wild-type and mutated putative binding sites of circLOC101928570 transcripts (Fig. 2D) and then cotransfected them with miR-150-3p/5p mimics or inhibitors into 293 T cells. Luciferase reporter assays showed that the luciferase activities of the circLOC101928570 wild-type reporter were significantly reduced when transfected with miR-150-3p/5p mimics compared with the control reporter or mutated luciferase reporter (Fig. 2E). However, the miR-150-3p/5p inhibitor significantly increased the luciferase signal of the wild-type circLOC101928570 reporter compared with the control reporter or the mutated luciferase reporter (Fig. 2F). qRT–PCR further confirmed that circLOC101928570 knockdown could increase the miR-150-5p level and that circLOC101928570 overexpression had the opposite effect in Jurkat cell lines (Fig. 2G). However, miR-150-3p/5p did not significantly influence circLOC101928570 levels (Fig. 2H). Moreover, RNA FISH assays revealed that circLOC101928570 and miR-150-5p were colocalized in the cytoplasm (Fig. 2I). These results showed that circLOC101928570 can bind to miR-150-5p.

### CircLOC101928570 decreased c-myb expression by sponging miR-150-5p

**Fig. 3:**
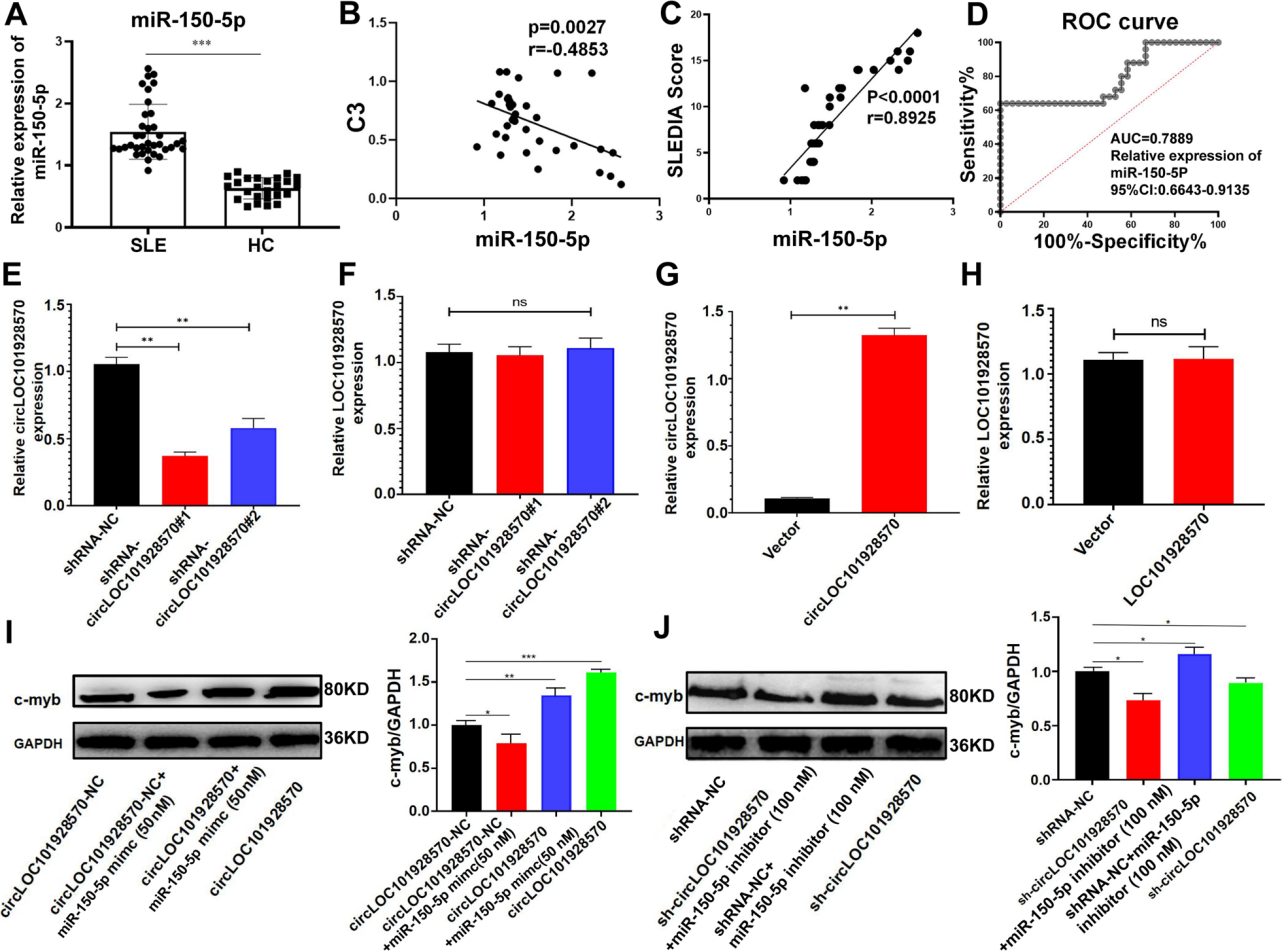
The expression of miR-150-5p in SLE. CircLOC101928570 decreased the c-myb expression **A** Expression of miR-150-5p in PBMCs from 36 patients with SLE and 25 healthy controls (HCs). **B**, **C** Correlation between the expression of miR-150-5p and the SLEDAI score or complement C3 level. **D** The ROC curve of relative miR-150-5p expression for the 36 different patients with SLE from 25 healthy controls. **E**, **F** Expression levels of circLOC101928570 and LOC101928570 in Jurkat cells treated with shRNA-NC, shRNA-circLOC101928570#1, or shRNA-circLOC101928570#2 lentivirus. **G**, **H** Expression levels of circLOC101928570 and LOC101928570 in Jurkat cells after transfection with circLOC101928570 lentivirus. **I**, **J** Western blot analysis of c-myb proteins after transfection with miR-150-5p mimics, circLOC101928570 overexpression plasmid, circLOC101928570-NC and the miR-150-5p inhibitors shRNA-circLOC101928570#1 and shRNA-NC in Jurkat cells. The results are represented as the mean ± SD (n = 3). NS: no significance, *P < 0.05, **P < 0.01, ***P < 0.001.

To explore the function of miR-150 in SLE, we then detected the expression of miR-150-5p using qRT–PCR in PBMCs obtained from 36 patients with SLE and 25 healthy controls. The results showed that the expression of miR-150-5p was upregulated in SLE (Fig. 3A). MiR-150-5p expression was also contrarily correlated with complement C3 levels (Fig. 3B). There was a strong positive correlation between miR-150-5p expression and the SLEDAI score in patients with SLE (Fig. 3C). To assess the diagnostic value of miR-150-5p in SLE, we also performed ROC curve analysis with the relative miR-150-5p expression in the 36 patients and 25 healthy controls (Fig. 3D). The AUC was 0.7889, and the 95% CI was 0.6643–0.9135. These results demonstrated that miR-150 was highly expressed in SLE patients and correlated with SLE.

It has been widely reported that c-myb is the target protein of miR-150. MiR-150 is highly expressed in mature lymphocytes, and c-myb is an important transcription factor for regulating lymphocyte development and participating in the pathogenesis of SLE [26–29]. To further study the role of circLOC101928570 in progression, we constructed two short hairpin RNAs (shRNA-circLOC101928570#1, shRNA-circLOC101928570#2). Jurkat cells with stable circLOC101928570 knockdown with lentiviral shRNA and circLOC101928570 overexpression plasmid with lentiviral were then established. We found that shRNA-circLOC101928570 successfully knocked down circLOC101928570 expression but had no effect on LOC101928570 mRNA expression in Jurkat cells (Fig. 3E, F). Similarly, circLOC101928570 was successfully overexpressed in Jurkat cells, while LOC101928570 mRNA expression had no obvious change (Fig. 3G, H). To explore whether circLOC101928570 could regulate c-myb by competitively binding with miR-150-5p, we transfected miR-150-5p mimics and circLOC101928570 overexpression plasmids into Jurkat cells. CircLOC101928570 overexpression increased the expression of c-myb, while transfected miR-150-5p mimics significantly attenuated the circLOC101928570-induced increase in the expression of c-myb (Fig. 3I). Downregulation of circLOC101928570 resulted in decreased expression of c-myb. Furthermore, transfection with the miR-150-5p inhibitor promoted the decreased expression of c-myb (Fig. 3J). These data demonstrated that circLOC101928570 regulates c-myb by competitively binding miR-150-5p to mediate the immune inflammatory response in SLE.

### CircLOC101928570 suppresses apoptosis

As a proto-oncogene, c-myb plays crucial roles in the processes of cell development, differentiation and apoptosis. Downregulated expression of c-myb will cause cell apoptosis [30–32]. Furthermore, previous studies have reported that PBMCs from individuals with SLE showed significantly decreased levels of c-myb [28, 29]. The expression of c-myb has a positive correlation with the number of immune complexes (ICs) and clinical disease activity [28]. Our study has shown that the downregulation of circLOC101928570 decreased c-myb expression by sponging miR-150-5p in SLE, suggesting that circLOC101928570 might be involved in SLE pathogenesis by regulating c-myb expression.

**Fig. 4:**
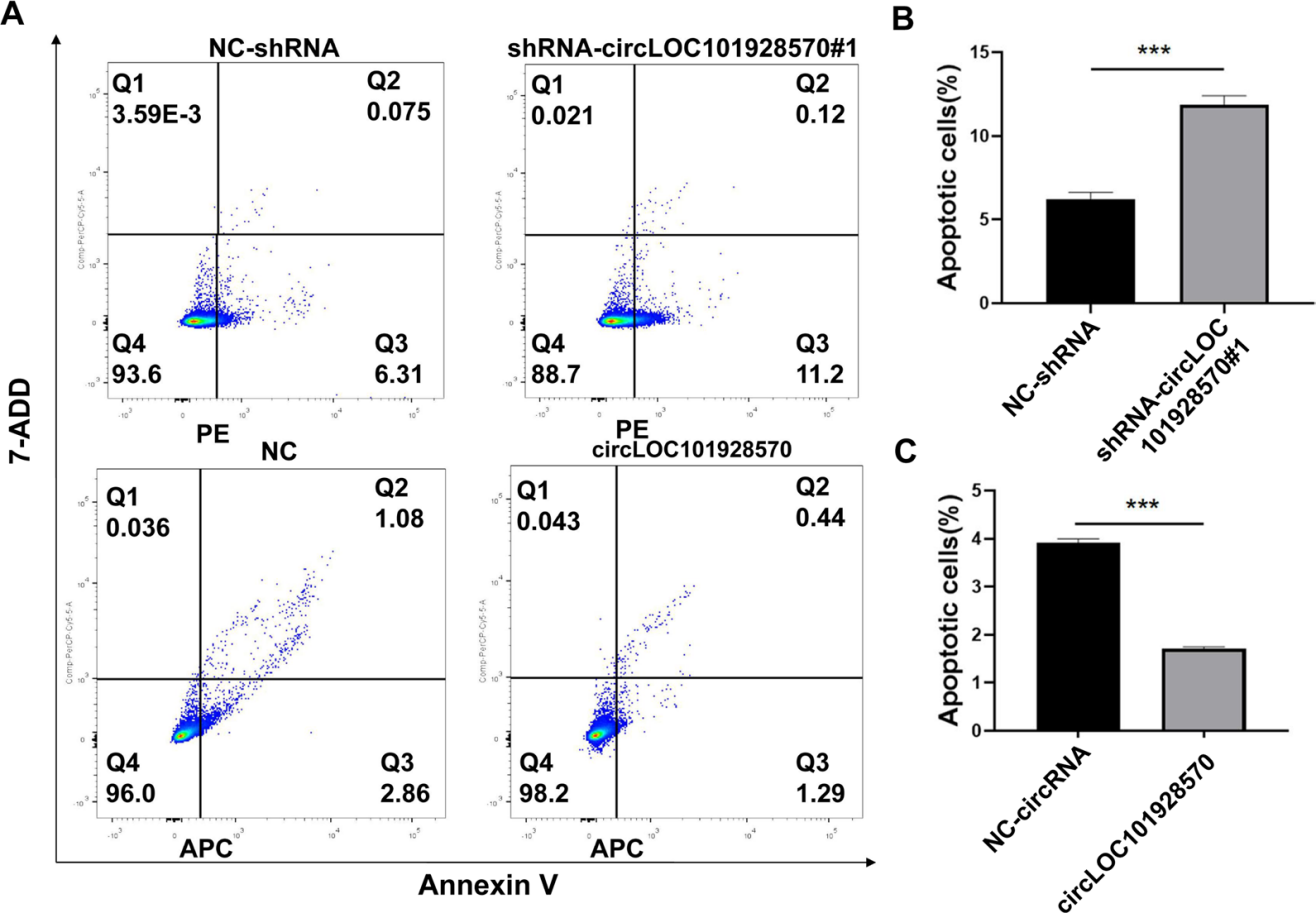
circLOC101928570 suppresses apoptosis. **A** Representative graphs of cell apoptosis determined by APC Annexin V and 7-AAD staining in Jurkat cells stably transfected with circLOC10192857 and negative control circRNA (NC-circRNA). PE Annexin V and 7-AAD staining in Jurkat cells stably transfected with shRNA-circLOC101928570#1 and negative control shRNA (NC-shRNA). **B**, **C** The percentage of apoptotic cells was significantly increased in the shRNA-circLOC101928570#1 group and significantly decreased in the circLOC101928570 group. The results are represented as the mean ± SD (n = 3). ***P < 0.001

To further explore the function of circLOC101928570, we examined cell apoptosis in stable Jurkat cells and found that the percentage of apoptotic cells was significantly increased in the shRNA-circLOC101928570#1 group compared with the shRNA-NC group (Fig. 4A, B). The proportion of apoptotic cells was lower in the circLOC101928570 overexpression group than in the NC group (Fig. 4A and C). These findings suggested that circLOC101928570 negatively regulated the early apoptosis of Jurkat cells. Identifying the factors contributing to the enhanced apoptosis of SLE T cells will deepen our understanding of SLE pathogenesis.

### C-myb transcriptionally regulates IL2RA expression by binding to IL2RA

**Fig. 5:**
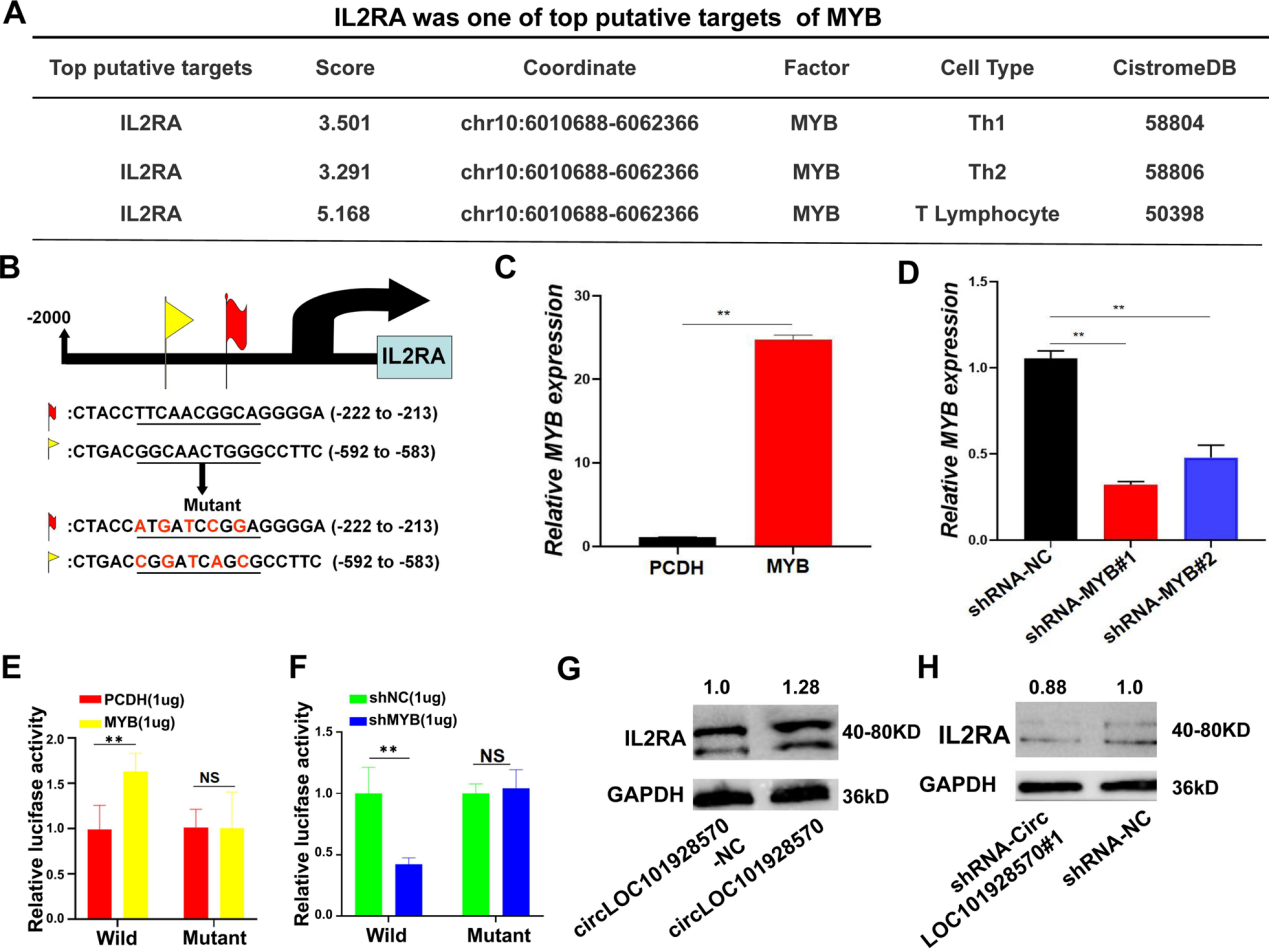
C-myb transcriptionally regulated IL2RA expression by binding to IL2RA. CircLOC101928570 suppresses IL2RA expression. **A** Prediction of the target gene of the transcription factor c-myb. **B** The binding sites of c-myb in the promoter of IL2RA are shown in a model. Schematic illustration of the sequences of the wild-type IL2RA promoter domain and mutant sequences in the binding sites of c-myb on the IL2RA promoter domain are shown. **C** Expression levels of MYB in Jurkat cells after transfection with the MYB plasmid. **D** Expression levels of MYB in 293 T cells treated with shRNA-NC, shRNA-MYB#1, or shRNA-MYB#2. **E**, **F** Relative luciferase activities were detected in 293 T cells after transfecting luciferase reporter plasmids with the wild-type IL2RA promoter domain or the mutant IL2RA promoter domain with pCDH, MYB, shRNA-NC, and shRNA-MYB#1. **G**, **H** Western blot analysis of IL2RA proteins in Jurkat cells transfected with circLOC101928570 overexpression plasmid, empty vectors, shRNA-NC, and shRNA-circLOC101928570#1. The results are represented as the mean ± SD (n = 3). NS: no significance, **P < 0.01

We then further explored the pathogenesis of circLOC101928570 involvement in SLE. Software-based prediction of transcription factor c-myb target genes (http://cistrome.org/db/#/) revealed that IL2RA might bind c-myb in Th1 and Th2 CD4^+^ T cells and T lymphocytes (Fig. 5A). CD4^+^ T lymphocytes are an important factor in the pathogenesis of SLE, mainly manifested by the immune imbalance of CD4^+^ T lymphocytes, and the differentiation of CD4^+^ T lymphocytes is regulated by IL-2 [33]. However, the specific mechanism remains to be clarified. Normal serum IL-2 levels are an important condition to maintain the normal function of CD4^+^ T lymphocytes, B cells and NK cells. As a transcription factor, c-myb may regulate target gene expression at the transcriptional level, thereby exerting biological functions. We analyzed the potential binding DNA sequence loop of c-myb and found two theoretical binding sites in the top 2000 nt of the promoter domain of the IL2RA gene (http://jaspar.genereg.net). Hence, we speculated that c-myb may regulate IL2RA expression at the transcriptional level. To confirm this supposition, a luciferase plasmid with the top 2000 nt of the promoter domain of the IL2RA gene (psicheck2-WT) and a luciferase plasmid with mutant sequences in both binding sites of the top 2000 nt of the promoter domain (psicheck2-Mutant) were generated (Fig. 5B). In addition, we designed two short hairpin RNAs (shRNA-MYB#1 and shRNA-MYB#2), and an MYB overexpression plasmid was constructed with a PCDH vector. We found that shRNA-MYB successfully knocked down MYB expression and that MYB was successfully overexpressed in 293 T cells (Fig. 5C and D). Luciferase reporter assays demonstrated that c-myb enhanced the luciferase activity of psicheck2-WT in a dose-dependent manner but not that of psicheck2-Mutant (Fig. 5E, F), suggesting that c-myb enhanced IL2RA expression by directly binding to the promoter domain of IL2RA. These data demonstrated that c-myb may suppress SLE progression by positively regulating IL2RA expression at the transcriptional level. To explore whether circLOC101928570 could regulate the expression of IL2RA, we transfected circLOC101928570 overexpression plasmid, circLOC101928570-NC, shRNA-circLOC101928570#1, and shRNA-NC into Jurkat cells. Upregulated circLOC101928570 increased the expression of IL2RA (Fig. 5G). Downregulation of circLOC101928570 resulted in the decreased expression of IL2RA (Fig. 5H). These data indicated the function of circLOC101928570 in regulating IL-2RA expression.

### CircLOC101928570 suppresses IL2RA expression in T cell subsets of SLE

**Fig. 6:**
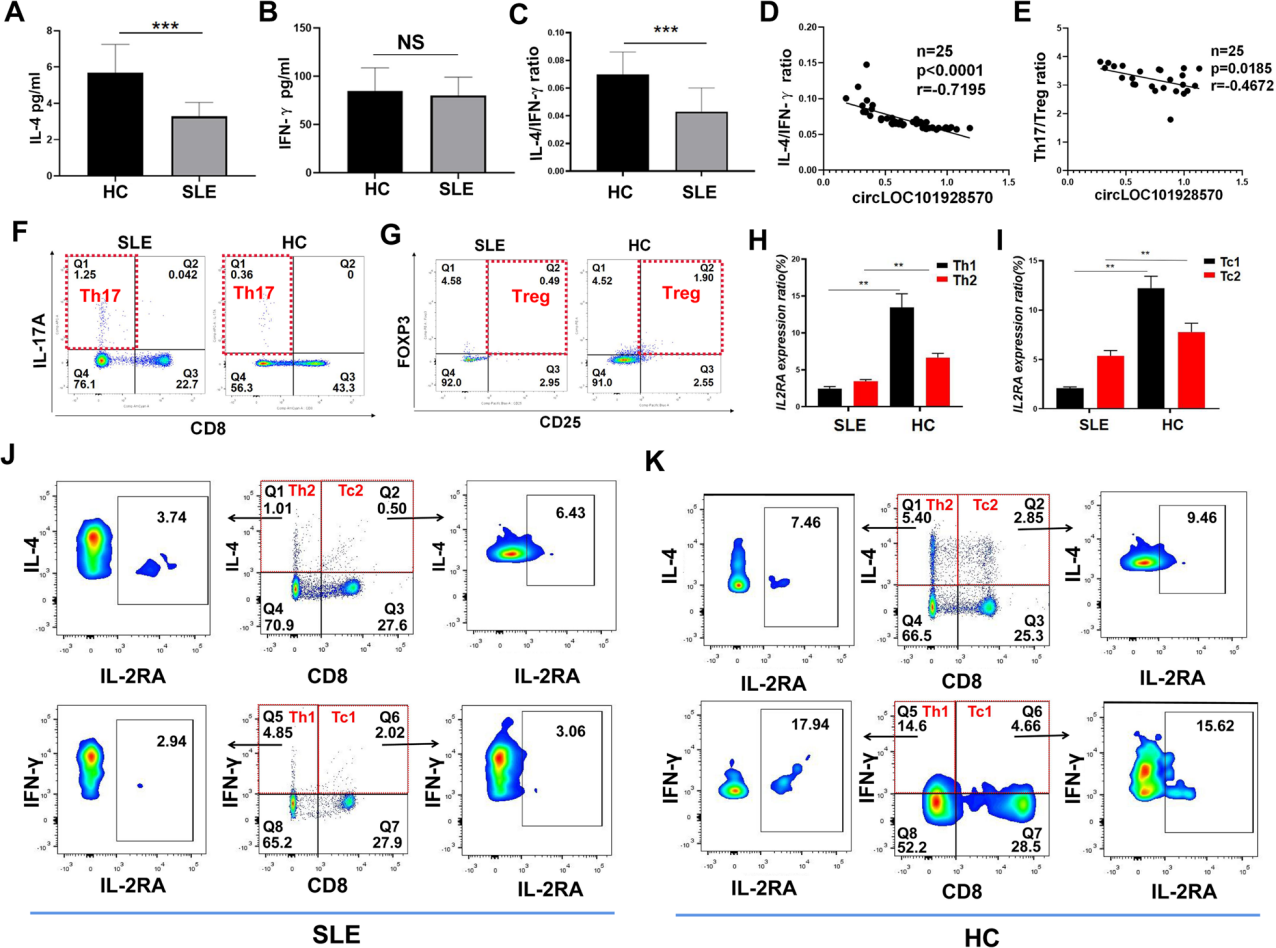
CircLOC101928570 suppresses IL2RA expression in T cell subsets of SLE. **A**–**C** Quantitative measurements of the IL-4, IFN-γ, and IL-4/IFN-γ levels in the serum from SLE patients and healthy controls by ELISA. **D**, **E** Correlation analysis of IL-4/IFN-γ and the Th17/Treg ratio with the expression of circLOC101928570 in SLE. **F**, **G** Representative flow cytometry results showing the percentages of Th17 and Treg cells in PBMCs from SLE patients compared with healthy controls. **H**, **I** The percentages of IL2RA Th1, Th2, Tc1 and Tc2 cells in PBMCs from SLE patients compared with those from healthy controls. **J**, **K** Representative flow cytometry results showing the percentages of IL2RAs among Th1, Th2, Tc1 and Tc2 cells in PBMCs from SLE patients compared with the healthy control group. The results are represented as the mean ± SD (n = 3). NS: no significance. **P < 0.01, ***p < 0.001

We analyzed the cell cytokine expression in the serum from 55 different SLE and 33 healthy groups by ELISA. IL-4 expression was decreased in the SLE group compared with the healthy group (Fig. 6A). There was no significant difference in IFN-γ expression in SLE patients compared with healthy controls (Fig. 6B). The IL-4/IFN-γ level in the SLE group was lower than that in the healthy group (Fig. 6C). The IL-4/IFN-γ ratio of SLE was negatively correlated with the expression of circLOC101928570 (Fig. 6D). Meanwhile, the Th17/Treg ratios negatively correlated with the expression of circLOC101928570 (Fig. 6E). We analyzed the Th17 and Treg percentages in PBMCs from the SLE and healthy groups by flow cytometry (Fig. 6F, G). The complete gating strategy regarding the flow cytometry for Th17 and Treg cells was shown in the Additional file 5: Figure S1-2. By linear regression analysis, the Th1/Th2 and Th17/Treg ratios were correlated with the expression of circLOC101928570. Next, we analyzed the expression of IL2RA on Th1, Th2, Tc1 and Tc2 cells in the T cell populations between SLE patients and healthy controls by flow cytometry. The results demonstrated that the expression of IL2RA on Th1, Th2, Tc1 and Tc2 cells in SLE patients was lower than that in healthy controls (Fig. 6H, I). We analyzed the percentages of IL2RAs among Th1, Th2, Tc1 and Tc2 cells in PBMCs from different SLE and healthy groups by flow cytometry (Fig. 6J, K). The complete gating strategy regarding the flow cytometry was shown in the Additional file 5: Figure S3. These data indicated that circLOC101928570 suppresses IL2RA expression in T cell subsets of SLE.

## Discussion

CircRNAs are a novel type of noncoding RNA that have multiple potential biological functions. Recently, an increasing number of studies have reported that circRNAs participate in the physiology and pathology of various diseases, and circRNAs have been identified as potential biomarkers for disease diagnosis or prognosis, including in autoimmune diseases. Several studies have revealed circRNAs as a potential clinical biomarker for SLE [34, 35]. Of note, the T cell-derived exosomes contain miRNAs and circRNAs, which can be transported between cells [36]. CircRNAs exert their biological functions through interactions with miRNAs or RNA binding proteins (RBPs) or as protein scaffolds, modulating gene transcription and protein translation [37, 38]. The recent discovery of thousands of circRNAs and their novel functions in gene expression regulation prompted us to investigate their roles in SLE. A misregulated circRNA-PKR-RNase L axis was found in SLE [39]. Zhang et al. demonstrated that hsa_circ_0012919 was associated with clinical variables and the abnormal DNA methylation present in SLE CD4^+^ T cells. It acts as a miRNA sponge for miR-125a-3p, regulating the gene expression of the protein targets RANTES and KLF13, which are involved in the physiology and pathophysiology of acute and chronic inflammatory processes [40]. Zhao et al. suggested that upregulated plasma circRNA_002453 levels in LN patients are associated with the severity of renal involvement and may also serve as a potential biomarker for LN patients [41]. circIBTK was downregulated in SLE and might regulate DNA demethylation and the AKT signaling pathway by binding to miR-29b in SLE [42]. These findings support the role of circRNAs in the pathophysiology of SLE.

In this study, we screened a downregulated circRNA named circLOC101928570 according to the results of RNA-seq and qRT–PCR analysis. We confirmed that circLOC101928570 expression was downregulated in SLE patients compared with healthy controls, and the expression of circLOC101928570 was correlated with the disease activity of SLE. Furthermore, we investigated the function and mechanism of circLOC101928570. Bioinformatics analysis showed that circLOC101928570 binds to miR-150-3p/5p. Luciferase reporter and RIP assays confirmed the direct interaction between circLOC101928570 and miR-150-5p, suggesting that circLOC101928570 functions as an “miRNA sponge” of miR-150-5p. Previous studies have demonstrated the pathogenic role of miR-150 in SLE. MiR-150 was identified to be positively correlated with chronicity scores and the expression of profibrotic proteins in lupus nephritis patients. Elevated miR-150 could target the antifibrotic protein SOCS1 with upregulated profibrotic proteins [23].

C-myb is an evolutionarily conserved miR-150 target, and studies have shown that the miR-150/c-myb interaction plays important roles in the differentiation of T cells and B cells, pressure overload-induced cardiac fibrosis, and the regulation of epithelial-mesenchymal transition (EMT) in ovarian cancer cells [24]. In our study, further molecular experiments demonstrated that circLOC101928570 increased c-myb expression by sponging miR-150-5p. Additionally, abnormal apoptosis and cytokine secretion of T cells are involved in the pathogenesis of SLE [43, 44]. We analyzed the role of circLOC101928570 in the apoptosis of Jurkat cells, and knockdown of circLOC101928570 led to increased levels of early apoptosis in Jurkat cells. Next, we identified that c-myb interacts with IL2RA by bioinformatics analysis and dual-luciferase reporter assay, which suggests that circRNAs are implicated in numerous posttranscriptional aspects of mRNA. Moreover, we found that circLOC101928570 expression was negatively correlated with the IL-4/IFN-γ ratio in SLE patients. The expression of IL2RA on Th1, Th2, Tc1 and Tc2 T cell populations in SLE patients was also significantly lower than that in healthy controls. This may show that circLOC101928570 may affect cytokine expression and the differentiation of T cells in SLE.

IL2RA is a subunit of the high-affinity receptor for interleukin-2 (IL-2). IL2RA plays a key role in the development and proliferation of functional T cells and selectively reduces the number of CD4^+^ and CD8^+^ T lymphocytes in the decidua in normal pregnancy [45]. The gene locus of IL2RA has been ascertained as a risk factor for a diverse series of autoimmune diseases, including SLE. The IL-2 pathway is critical for the maintenance of immune homeostasis. IL-2 signaling plays a role in activation-induced cell death and is vital to regulatory T cell homeostasis [46, 47]. CD4^+^ T cell-derived IL-2 is essential for CD8^+^ T cell responses to noninflammatory conditions, IL-2 helps CD8^+^ T cells initiate responses to pathogens, and protective memory is also required to stimulate CD8^+^ T cells through IL-2 during initiation [48]. Furthermore, the efficacy of adoptive immunotherapy of CD8^+^ T cells may be influenced by the opposite differentiation programs of IL-2 and IL-21 [49]. According to the correlation between Th1/Th2 and the Tc1/Tc2 ratio, serum sIL-2Rɑ levels may reflect the immune response status [50]. In our study, we found that the expression levels of IL2RA on Th1, Th2, Tc1 and Tc2 T cell populations in SLE patients were also significantly lower than in healthy controls. C-myb transcriptionally regulates IL2RA expression by binding to IL2RA. These data indicated that circLOC101928570 may influence IL2RA expression in T cell subsets of SLE.

There are accumulating examples of circRNAs acting as miRNA sponges, thereby influencing the target mRNAs. Moreover, several miRNAs regulating pathogenesis and involved in the apoptotic pathway have shown therapeutic potential in SLE [51, 52]. Whether and to what extent circRNA expression contributes to the dysregulated miRNA profile in SLE are questions that remain to be solved. Our study demonstrated that the functional dysregulations, including apoptosis, seen in Jurkat cells may be related to circLOC101928570 regulation of relevant transcripts. The signaling pathway of apoptosis correlated with circLOC101928570 needs further exploration. In addition, our study showed that circLOC101928570 acted as a miR-150-5p sponge to modulate the activation of immune inflammatory responses mediated by the c-myb/IL2RA pathway. We found that circLOC101928570 influences IL2RA expression in Th1, Th2, Tc1 and Tc2 T cell subsets of SLE. How the low expression of IL2RA in the CD4^+^ and CD8^+^ T cell subsets participates in the pathogenesis of SLE is not clear. We suspect that IL2RA in different CD4^+^ and CD8^+^ T cell subsets will influence binding with IL-2. Defective IL-2 production is one of many factors involved in the immune dysregulation responsible for SLE. Decreased IL-2 production in SLE patients leads to many immune defects, such as decreased production, decreased activation-induced cell death (AICD) and decreased cytotoxicity [53]. A follow-up study to elucidate a deeper understanding of circLOC101928570 on the defective function of IL-2 is still needed.

In this study, we only used CD3 and CD8 antibodies to identify CD4^+^ T cells (CD3^+^CD8^−^) and CD8^+^ T cells (CD3^+^CD8^+^). However, it has been reported that CD4 and CD8 double-negative T cells are involved in pathogenesis by producing IL-17 and IFN-γ [54]. This means that CD3^+^CD8^−^ T cells could not be taken as *bona fide* CD4^+^ T cells, although they could roughly reflect the frequency of CD4^+^ T cells, and more importantly, they could be used to compare the cell frequency difference between the two groups. Moreover, the circulating percentage of memory vs naive T cells, and other innate cell types also need to check in future studies. In addition, the changes in the metabolisms of the PBMCs across the two groups, the apoptotic gene/protein expression in the Jurkat cells, and the downstream pathways or genes in the apoptosis of the immune cells also requires our follow-up further verification by biological experiments. More importantly, the experiments in vivo are needed to further verify the role of circLOC101928570 in SLE. This should be the limitation of this study. Together, the exact mechanisms of the pathogenesis and development of SLE induced by circLOC101928570 need to be further investigated.

## Conclusions

**Fig. 7:**
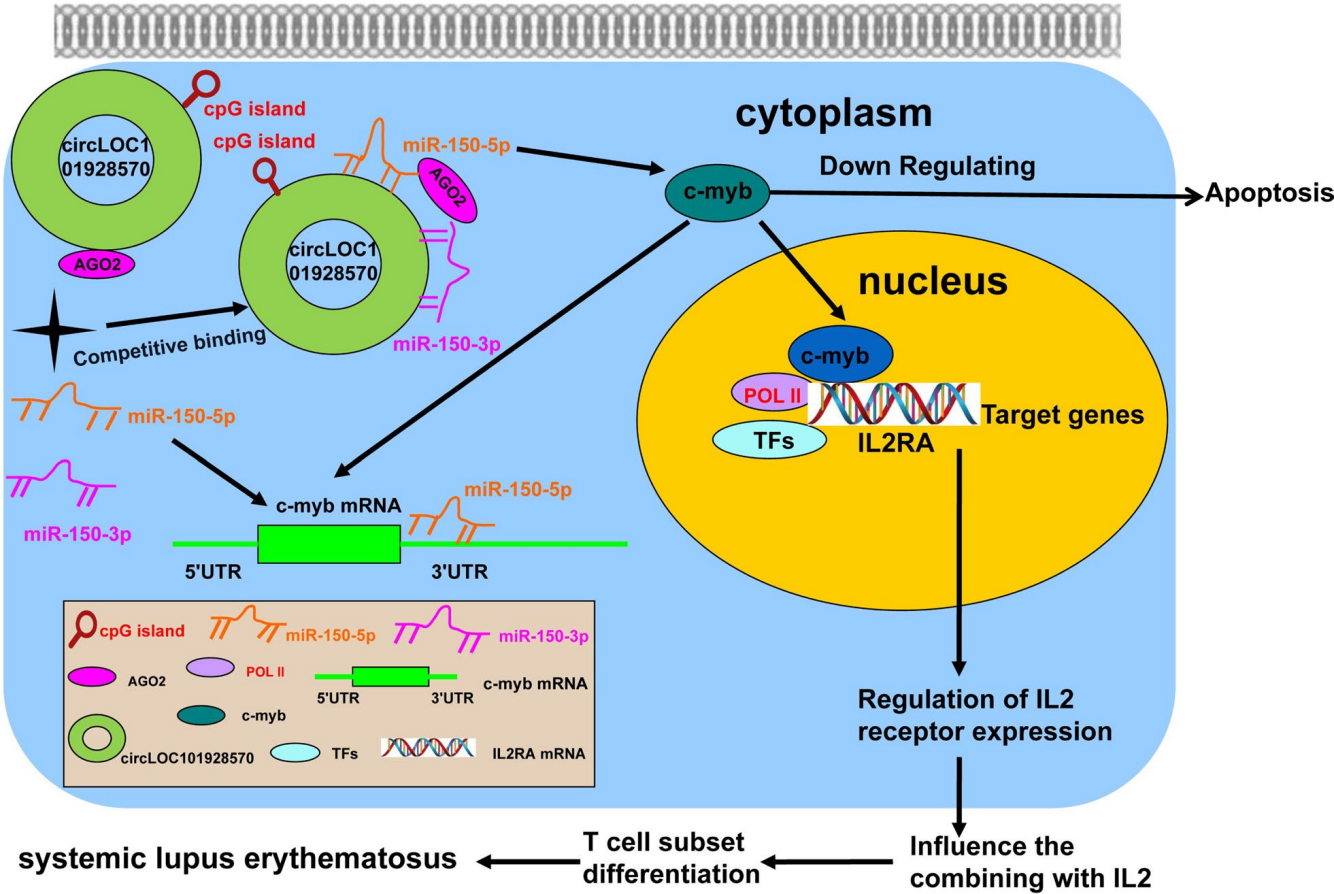
Schematic depicting the dominant function of circLOC101928570. CircLOC101928570 acted as a miR-150-5p sponge to modulate the activation of immune inflammatory responses mediated by the c-myb/IL2RA pathway. CircLOC101928570 knockdown enhanced apoptosis

In conclusion, we provided the first evidence that the expression of circLOC101928570 is significantly decreased in SLE patients. Further experiments demonstrated that circLOC101928570 acted as an miR-150-5p sponge to modulate the activation of immune inflammatory responses mediated by the c-myb/IL2RA pathway. CircLOC101928570 negatively regulates apoptosis and influences the regulation of IL2RA expression in T cell subsets of SLE. Our results suggested that circLOC101928570 might be a biomarker for SLE. The regulatory network involving the circLOC101928570/miR-150-5p/c-myb/IL2RA pathway might provide new insight into the potential mechanisms of the pathogenesis and development of SLE (Fig. 7).

## Supplementary Information

**Additional file 1: Table S1. **Detailed information on the SLE patients.

**Additional file 2: Table S2. **The sequences of the primers used for qRT–PCR.

**Additional file 3: Table S3. **The shRNA sequences used in this study.

**Additional file 4: Table S4. **Identified and selected upregulated circRNAs and downregulated circRNAs (|fold change| >2, P < 0.01) between healthy controls and SLE patients by circRNA RNA-seq data.

**Additional file 5: Figure S1. **Gating strategy for the detection of Th17 cells. Figure represents the result of (A) the patient with SLE compared to (B) the healthy control. Lymphocyte population was gated from PBMCs according to forward scatter area (FSC-A) characteristics and side scatter area (SSC-A) characteristics. Discrimination of single cells was performed by plotting forward scatter height (FSC-H) against forward scatter area (FSC-A). Gating strategy to discriminate T cell populations was set using CD3-antibody. The CD3^+^CD8^-^IL17A^+^ cells (namely Th17) were then separated from the gated T lymphocytes. **Figure S2. **Gating strategy for the detection of Treg cells. Figure represents the result of (A) the patient with SLE compared to (B) the healthy control. Lymphocyte population was gated from PBMCs according to forward scatter area (FSC-A) and side scatter area (SSC-A) characteristics. Discrimination of single cells was performed by plotting forward scatter height (FSC-H) against forward scatter area (FSC-A). Gating strategy to discriminate CD4^+^T cell populations was set using CD3-antibody and CD4-antibody. Treg cells (CD3^+^CD4^+^CD25^+^FOXP3^+^) were then gated from CD4^+^T cells. **Figure S3. **Gating strategy for the detection of Th1, Th2, Tc1 and Tc2 cells. Representative examples of (A) the patient with SLE compared to (B) the healthy control. Lymphocyte population was gated from PBMCs according to forward scatter (FSC) and side scatter (SSC) characteristics. Discrimination of single cells was performed by plotting forward scatter height (FSC-H) against forward scatter area (FSC-A). Gating strategy to discriminate T cell populations was set using CD3-antibody. Th1 (CD3^+^CD8^-^IL-4^high^), Th2 (CD3^+^CD8^-^IFN-γ^high^), Tc1 (CD3^+^CD8^+^IFN-γ^high^) and Tc2 (CD3^+^CD8^+^IL-4^high^) cells were then gate from T cell population by using the indicated antibodies. IL2RA expression in Th1, Th2, Tc1 and Tc2 subpopulations from SLE patients and healthy controls was detected by staining with pacific blue labeled anti-human IL2RA-antibody.

## Data Availability

The original contributions presented in the study are included in the article/Supplementary Material, and further inquiries can be directed to the corresponding author/s.

## Abbreviations

SLE: Systemic lupus erythematosus
circRNAs: Circular RNAs
PBMCs: Peripheral blood mononuclear cells
MiR-150: MicroRNA-150
miRNAs: MicroRNAs
SLEDAI: Systemic lupus erythematosus disease activity index
ceRNA: Competing endogenous RNAs
ROC: Receiver operating characteristic
AUC: Area under the curve
MYB: MYB Proto-oncogene, transcription factor
IL2RA: Interleukin 2 receptor subunit alpha
IgG: Immunoglobulin G
cDNA: Complementary DNA
WT: Wild-type
EMT: Epithelial-mesenchymal transition
RBPs: RNA-binding proteins
ELISA: Enzyme-linked immunosorbent assay
FISH: Fluorescence in situ hybridization
qRT–PCR: Quantitative real-time polymerase chain reaction
RIP: RNA-binding protein immunoprecipitation
AGO2: Argonaut 2
WB: Western blotting
