# Supplementary material for: Circular RNA circLOC101928570 suppresses systemic lupus erythematosus progression by targeting the miR-150-5p/c-myb axis
Source: J Transl Med. 2022 Nov 26;20:547. doi: 10.1186/s12967-022-03748-2 (PMC9701435; doi:10.1186/s12967-022-03748-2)
Supplement: Supplementary file 2 — Additional file 2: Table S2. The sequences of the primers used for qRT–PCR. [file 12967_2022_3748_MOESM2_ESM.doc]

**Supplementary Table 2 Primers used in quantitative real-time PCR experiment**

| **RNAs** | **Primers** |
| --- | --- |
| **circLOC101928570** | **F:5'ACTCTCAAGTCCCCTCCATCT 3'**  **R:5'GCAAGGCTCATACCTTGGTACA3'** |
| **LOC101928570** | **F:5'CCTCCTTGTGAGTTGCCACTTCC3'**  **R:5'AGCCACTGATGAGAACCTTCTTGC3'** |
| **β-Actin** | **F:5'GGTGAGCTGCGAGAATAGCC3'**  **R:5'CTCCGACCAGTGTTTGCCTT3'** |
| **U6** | **F:5'TCGCTTCGGCAGCACATA3'**  **R:5'TTTGCGTGTCATCCTTGC3'** |
| **Reverse transcription primer** | **5'GTCGTATCCAGTGCGTGTCGTGGAGTCGGCAATTGCACTGGATACGACXXXXXXXX3'** |
| **miR-335-3p** | **F:5'GGGTTTTTCATTATTGCT3'**  **R:5'CAGTGCGTGTCGTGGAGT3'** |
| **miR-498** | **F:5'GGGTTTCAAGCCAGGGGGCG3'**  **R:5'CAGTGCGTGTCGTGGAGT3'** |
| **miR-30a-3p** | **F:5'GGGCTTTCAGTCGGATGTT3'**  **R:5'CAGTGCGTGTCGTGGAGT3'** |
| **miR-3913-3p** | **F:5'GGGAGACATCAAGATCAGT3'**  **R:5'CAGTGCGTGTCGTGGAGT3'** |
| **miR-150-3p** | **F:5'GGGCTGGTACAGGCCTGGG3'**  **R:5'CAGTGCGTGTCGTGGAGT3'** |
| **miR-150-5p** | **F:5'GGGTCTCCCAACCCTTGTA3'**  **R:5'CAGTGCGTGTCGTGGAGT3'** |
| **miR-3924** | **F:5'GGGATATGTATATGTGACT3'**  **R:5'CAGTGCGTGTCGTGGAGT3'** |
| **miR-3977** | **F:5'GGGGTGCTTCATCGTAATTA3'**  **R:5'CAGTGCGTGTCGTGGAGT3'** |
| **miR-34a-3p** | **F:5'GGGCAATCAGCAAGTATAC3'**  **R:5'CAGTGCGTGTCGTGGAGT3'** |
| **miR-545-5p** | **F:5'GGGTCAGTAAATGTTTATT3'**  **R:5'CAGTGCGTGTCGTGGAGT3'** |
