# Supplementary material for: Circular RNA circLOC101928570 suppresses systemic lupus erythematosus progression by targeting the miR-150-5p/c-myb axis
Source: J Transl Med. 2022 Nov 26;20:547. doi: 10.1186/s12967-022-03748-2 (PMC9701435; doi:10.1186/s12967-022-03748-2)
Supplement: Supplementary file 3 — Additional file 3: Table S3. The shRNA sequences used in this study. [file 12967_2022_3748_MOESM3_ESM.doc]

**Supplementary Table 3 Sequences used in shRNA constructed**

| **shRNAs** | **Sequences** | **target sequences** |
| --- | --- | --- |
| **shRNA-NC** | **5’-CCGGGCACTACCAGAGCTAACTCAGTTCAAGAGACTGAGTTAGCTCTGGTAGTGCTTTTTTGGTACC-3’** | **5’-GCACTACCAGAGCTAACTCAG-3’** |
| **shRNA-MYB#1** | **5’-CCGGGCGGCTGAATAGGTTGCTTGTTTCAAGAGAACAAGCAACCTATTCAGCCGCTTTTTTGGTACC-3’** | **5’-GCGGCTGAATAGGTTGCTTGT-3’** |
| **shRNA-MYB#2** | **5'-CCGGGCACACGACAGAGATCTTTCCTTCAAGAGAGGAAAGATCTCTGTCGTGTGCTTTTTTGGTACC-3'** | **5’-GCACACGACAGAGATCTTTCC-3’** |
| **shRNA-circLOC101928570#1** | **5’-CCGGGCCTTGCAAGAAGACATACATTTCAAGAGAATGTATGTCTTCTTGCAAGGCTTTTTTGGTACC-3’** | **5’-GCCTTGCAAGAAGACATACAT-3’** |
| **shRNA-circLOC101928570#2** | **5'-CACCGGGATAATGCATTGGGTATAATTCAAGAGATTATACCCAATGCATTATCCCTTTTTTGGTACC-3’** | **5’-GGGATAATGCATTGGGTATAA-3’** |
