# Supplementary material for: Circular RNA circLOC101928570 suppresses systemic lupus erythematosus progression by targeting the miR-150-5p/c-myb axis
Source: J Transl Med. 2022 Nov 26;20:547. doi: 10.1186/s12967-022-03748-2 (PMC9701435; doi:10.1186/s12967-022-03748-2)
Supplement: Supplementary file 4 — Additional file 4: Table S4. Identified and selected upregulated circRNAs and downregulated circRNAs (|fold change| >2, P < 0.01) between healthy controls and SLE patients by circRNA RNA-seq data. [file 12967_2022_3748_MOESM4_ESM.doc]

**Supplementary T**able 4 Top upregulated and downregulated aberrantly expressed circRNAs in SLE PBMCs.

| **GeneName** | **Style** | | **Length** | | **Log2FC** | | | **FDR** | **start** | | **end** | | | **strand** |
| --- | --- | --- | --- | --- | --- | --- | --- | --- | --- | --- | --- | --- | --- | --- |
| **MYBL1** | | **down** | | **1705** | | **-5.115477217** | **1.95974E-08** | | | **66572481** | | **66602523** | **-** | |
| **PTPN22** | | **down** | | **1429** | | **-5.078002512** | **3.74294E-10** | | | **113833110** | | **113855049** | **-** | |
| **EML5** | | **down** | | **470** | | **-4.700439718** | **0.012294472** | | | **88712270** | | **88715195** | **-** | |
| **MCTP2** | | **down** | | **275** | | **-4.415037499** | **0.015566341** | | | **94440175** | | **94458246** | **+** | |
| **LOC101928570** | | **down** | | **435** | | **-4.321928095** | **0.006856743** | | | **77271339** | | **77273307** | **-** | |
| **C6orf106** | | **down** | | **548** | | **-4.289506617** | **2.47287E-06** | | | **34606554** | | **34646798** | **-** | |
| **CCSER2** | | **down** | | **450** | | **-4.169925001** | **0.01281405** | | | **84417770** | | **84438707** | **+** | |
| **AMY2B** | | **down** | | **1307** | | **-4.078002512** | **0.000131076** | | | **103565434** | | **103575540** | **+** | |
| **R3HDM1** | | **down** | | **722** | | **-4.029747343** | **1.0622E-05** | | | **135631717** | | **135639122** | **+** | |
| **NCOA4** | | **down** | | **1218** | | **-4** | **0.025516196** | | | **46010222** | | **46013639** | **-** | |
| **ERBB2** | | **down** | | **939** | | **-3.91753784** | **0.011777322** | | | **39709812** | | **39715939** | **+** | |
| **DTHD1** | | **down** | | **1208** | | **-3.874469118** | **0.028977807** | | | **36290372** | | **36308493** | **+** | |
| **na** | | **down** | | **1687** | | **-3.841302254** | **0.030318611** | | | **69831052** | | **69832739** | **-** | |
| **NFYB** | | **down** | | **360** | | **-3.841302254** | **0.037961444** | | | **104120399** | | **104123423** | **-** | |
| **MILR1** | | **down** | | **693** | | **-3.736965594** | **0.025042574** | | | **64457399** | | **64467645** | **+** | |
| **TBC1D31** | | **down** | | **2647** | | **-3.736965594** | **0.043910305** | | | **123077110** | | **123144855** | **+** | |
| **DDX17** | | **down** | | **451** | | **-3.736965594** | **0.048582429** | | | **38498084** | | **38501280** | **-** | |
| **na** | | **down** | | **7265** | | **-3.678071905** | **0.042578824** | | | **85067126** | | **85074391** | **+** | |
| **CDC123** | | **down** | | **480** | | **-3.473931188** | **0.036945381** | | | **12215739** | | **12238485** | **+** | |
| **PCF11** | | **down** | | **4936** | | **-3.415037499** | **0.045826411** | | | **83161326** | | **83184908** | **+** | |
| **PIK3CA** | | **down** | | **332** | | **-3.378511623** | **0.008193713** | | | **179203543** | | **179204588** | **+** | |
| **EPS15** | | **down** | | **479** | | **-3.273018494** | **0.003381128** | | | **51440346** | | **51448135** | **-** | |
| **MKLN1** | | **down** | | **535** | | **-3.187627003** | **0.000260339** | | | **131387119** | | **131399433** | **+** | |
| **GSDMB** | | **down** | | **254** | | **-3.169925001** | **0.039352613** | | | **39908957** | | **39909924** | **-** | |
| **YPEL1** | | **down** | | **1061** | | **-3.169925001** | **0.039352613** | | | **21700975** | | **21710908** | **-** | |
| **ZNF362** | | **down** | | **334** | | **-3.152003093** | **0.042131419** | | | **33280123** | | **33280457** | **+** | |
| **RABEP1** | | **down** | | **315** | | **-3.078002512** | **0.029639018** | | | **5346789** | | **5350629** | **+** | |
| **DDX6** | | **down** | | **494** | | **-3.078002512** | **0.029639018** | | | **118758773** | | **118765355** | **-** | |
| **ERI3** | | **down** | | **547** | | **-3.058893689** | **0.02609448** | | | **44308309** | | **44339322** | **-** | |
| **GDI2** | | **down** | | **208** | | **-3** | **0.023058666** | | | **5796762** | | **5800705** | **-** | |
| **MYBL1** | | **down** | | **1038** | | **-2.959358016** | **0.041622277** | | | **66572481** | | **66593194** | **-** | |
| **ARAP2** | | **down** | | **582** | | **-2.906890596** | **0.021494335** | | | **36210389** | | **36214480** | **-** | |
| **MAN2A1** | | **down** | | **700** | | **-2.830074999** | **0.016156201** | | | **109713519** | | **109755456** | **+** | |
| **HIF1A** | | **down** | | **231** | | **-2.807354922** | **0.020678157** | | | **61721508** | | **61721823** | **+** | |
| **ESYT2** | | **down** | | **431** | | **-2.777607579** | **0.00743418** | | | **158773340** | | **158798076** | **-** | |
| **HNRNPC** | | **down** | | **401** | | **-2.746551643** | **0** | | | **21230318** | | **21234229** | **-** | |
| **SNRK** | | **down** | | **757** | | **-2.744161096** | **2.5315E-05** | | | **43299753** | | **43303792** | **+** | |
| **UXS1** | | **down** | | **197** | | **-2.736965594** | **0.004596197** | | | **106158057** | | **106166083** | **-** | |
| **SEC63** | | **down** | | **830** | | **-2.454565863** | **0.034825338** | | | **107904628** | | **107924932** | **-** | |
| **FOXJ3** | | **down** | | **512** | | **-2.222392421** | **0.036709282** | | | **42265114** | | **42278784** | **-** | |
| **SNX1** | | **down** | | **156** | | **-2.152003093** | **0.040654711** | | | **64112572** | | **64123546** | **+** | |
| **SLX4IP** | | **down** | | **211** | | **-2.128733314** | **0.018537476** | | | **10556230** | | **10560820** | **+** | |
| **ASXL1** | | **down** | | **195** | | **-2.097847323** | **0.000929974** | | | **32366383** | | **32369123** | **+** | |
| **TRNT1** | | **down** | | **460** | | **-2.019899557** | **0.029918387** | | | **3137259** | | **3144710** | **+** | |
| **STIL** | | **up** | | **1084** | | **2.044394119** | **0.018964881** | | | **47280240** | | **47282459** | **-** | |
| **VCAN** | | **up** | | **5262** | | **2.118941073** | **0.018067556** | | | **83537006** | | **83542268** | **+** | |
| **FANCL** | | **up** | | **444** | | **2.214319121** | **0.001945658** | | | **58198593** | | **58232112** | **-** | |
| **EPSTI1** | | **up** | | **375** | | **2.237039197** | **1.10613E-08** | | | **42953947** | | **42970670** | **-** | |
| **EMILIN2** | | **up** | | **1926** | | **2.276124405** | **2.70512E-07** | | | **2890560** | | **2892486** | **+** | |
| **FAM126A** | | **up** | | **654** | | **2.614709844** | **0.049095633** | | | **22976209** | | **22991139** | **-** | |
| **ARID1B** | | **up** | | **286** | | **2.672425342** | **0.002888065** | | | **157036834** | | **157084905** | **+** | |
| **ASH1L** | | **up** | | **844** | | **2.717412797** | **0.024980025** | | | **155438326** | | **155459898** | **-** | |
| **PGAP1** | | **up** | | **310** | | **2.906890596** | **0.033748199** | | | **196890827** | | **196898369** | **-** | |
| **POLI** | | **up** | | **291** | | **2.935459748** | **0.016269535** | | | **54271359** | | **54274090** | **+** | |
| **ATP8A1** | | **up** | | **398** | | **3.087462841** | **0.046499712** | | | **42485495** | | **42507154** | **-** | |
| **SLC15A4** | | **up** | | **296** | | **3.169925001** | **0.019492228** | | | **128814774** | | **128815070** | **-** | |
| **PRKCA** | | **up** | | **18266** | | **3.169925001** | **0.039913298** | | | **66496200** | | **66514466** | **+** | |
| **WDFY1** | | **up** | | **335** | | **3.209453366** | **0.018754421** | | | **223884647** | | **223895630** | **-** | |
| **ICA1** | | **up** | | **658** | | **3.209453366** | **0.028129238** | | | **8218304** | | **8236005** | **-** | |
| **RSU1** | | **up** | | **489** | | **3.247927513** | **0.020596844** | | | **16752538** | | **16782084** | **-** | |
| **MTDH** | | **up** | | **499** | | **3.321928095** | **0.042272963** | | | **97686667** | | **97691188** | **+** | |
| **ADCY9** | | **up** | | **369** | | **3.687462841** | **0.044939241** | | | **3979115** | | **3983440** | **-** | |
| **KIAA0922** | | **up** | | **283** | | **3.392317423** | **0.010872712** | | | **153626146** | | **153632838** | **+** | |
| **KPNB1** | | **up** | | **528** | | **3.392317423** | **0.02747368** | | | **47663088** | | **47668410** | **+** | |
| **PIAS1** | | **up** | | **359** | | **3.426264755** | **0.013329489** | | | **68141945** | | **68146700** | **+** | |
| **RALBP1** | | **up** | | **294** | | **3.672425342** | **0.024350113** | | | **9524593** | | **9525851** | **+** | |
| **GARS** | | **up** | | **254** | | **3.709658248** | **0.016824184** | | | **30621392** | | **30622462** | **+** | |
| **CBFB** | | **up** | | **361** | | **3.754887502** | **0.041641745** | | | **67036638** | | **67082339** | **+** | |
| **LYST** | | **up** | | **664** | | **3.794415866** | **0.046897945** | | | **235677090** | | **235697272** | **-** | |
| **BARD1** | | **up** | | **1261** | | **4.06608919** | **9.10507E-05** | | | **214767481** | | **214797117** | **-** | |
