# Supplementary material for: Circular RNA circLOC101928570 suppresses systemic lupus erythematosus progression by targeting the miR-150-5p/c-myb axis
Source: J Transl Med. 2022 Nov 26;20:547. doi: 10.1186/s12967-022-03748-2 (PMC9701435; doi:10.1186/s12967-022-03748-2)
Supplement: Supplementary file 5 — Additional file 5: Figure S1. Gating strategy for the detection of Th17 cells. Figure represents the result of (A) the patient with SLE compared to (B) the healthy control. Lymphocyte population was gated from PBMCs according to forward scatter area (FSC-A) characteristics and side scatter area (SSC-A) characteristics. Discrimination of single cells was performed by plotting forward scatter height (FSC-H) against forward scatter area (FSC-A). Gating strategy to discriminate T cell populations was set using CD3-antibody. The CD3+CD8-IL17A+ cells (namely Th17) were then separated from the gated T lymphocytes. Figure S2. Gating strategy for the detection of Treg cells. Figure represents the result of (A) the patient with SLE compared to (B) the healthy control. Lymphocyte population was gated from PBMCs according to forward scatter area (FSC-A) and side scatter area (SSC-A) characteristics. Discrimination of single cells was performed by plotting forward scatter height (FSC-H) against forward scatter area (FSC-A). Gating strategy to discriminate CD4+T cell populations was set using CD3-antibody and CD4-antibody. Treg cells (CD3+CD4+CD25+FOXP3+) were then gated from CD4+T cells. Figure S3. Gating strategy for the detection of Th1, Th2, Tc1 and Tc2 cells. Representative examples of (A) the patient with SLE compared to (B) the healthy control. Lymphocyte population was gated from PBMCs according to forward scatter (FSC) and side scatter (SSC) characteristics. Discrimination of single cells was performed by plotting forward scatter height (FSC-H) against forward scatter area (FSC-A). Gating strategy to discriminate T cell populations was set using CD3-antibody. Th1 (CD3+CD8-IL-4high), Th2 (CD3+CD8-IFN-γhigh), Tc1 (CD3+CD8+IFN-γhigh) and Tc2 (CD3+CD8+IL-4high) cells were then gate from T cell population by using the indicated antibodies. IL2RA expression in Th1, Th2, Tc1 and Tc2 subpopulations from SLE patients and healthy controls was detected by staining with pacific blue [file 12967_2022_3748_MOESM5_ESM.docx]

**Supplementary Figures**

**Additional file 5: Figure S1**


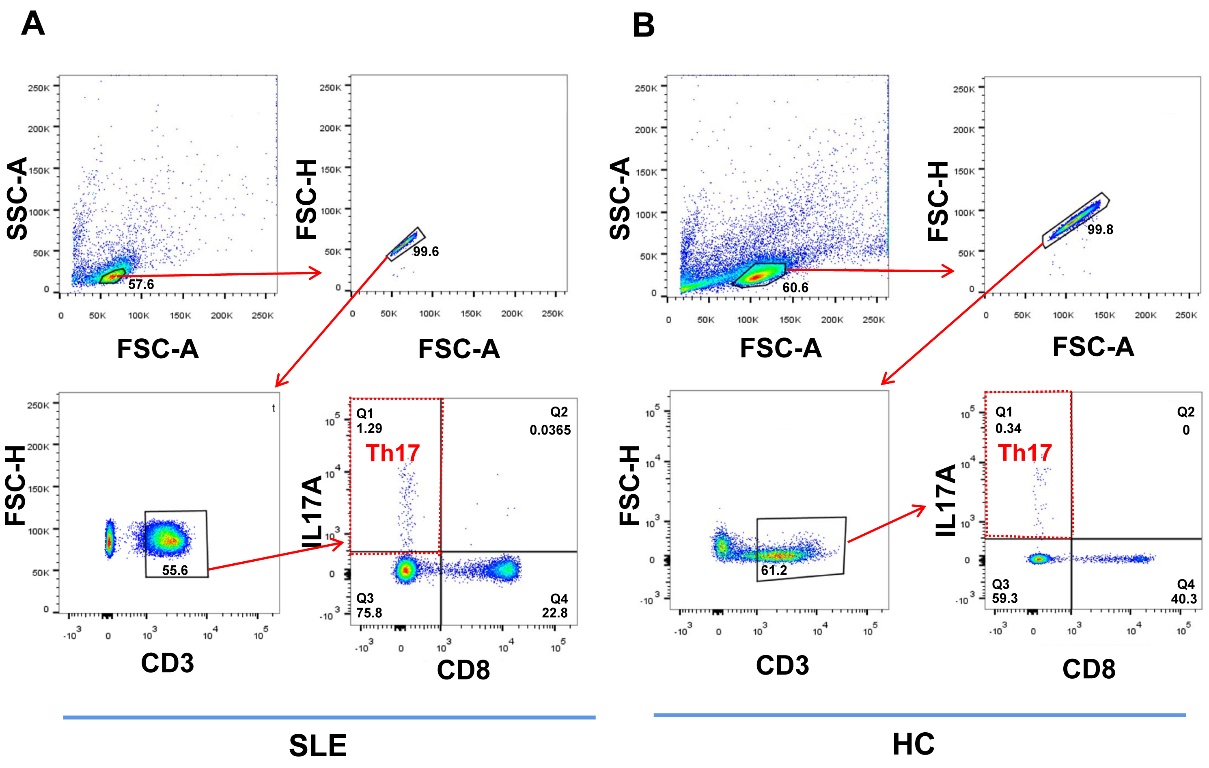


**Figure S1:** **Gating strategy for the detection of Th17 cells.**

Figure represents the result of **(A)** the patient with SLE compared to **(B)** the healthy control. Lymphocyte population was gated from PBMCs according to forward scatter area (FSC-A) characteristics and side scatter area (SSC-A) characteristics. Discrimination of single cells was performed by plotting forward scatter height (FSC-H) against forward scatter area (FSC-A). Gating strategy to discriminate T cell populations was set using CD3-antibody. The CD3^+^CD8^-^IL17A^+^ cells (namely Th17) were then separated from the gated T lymphocytes.

**Additional file 5: Figure S2**

**
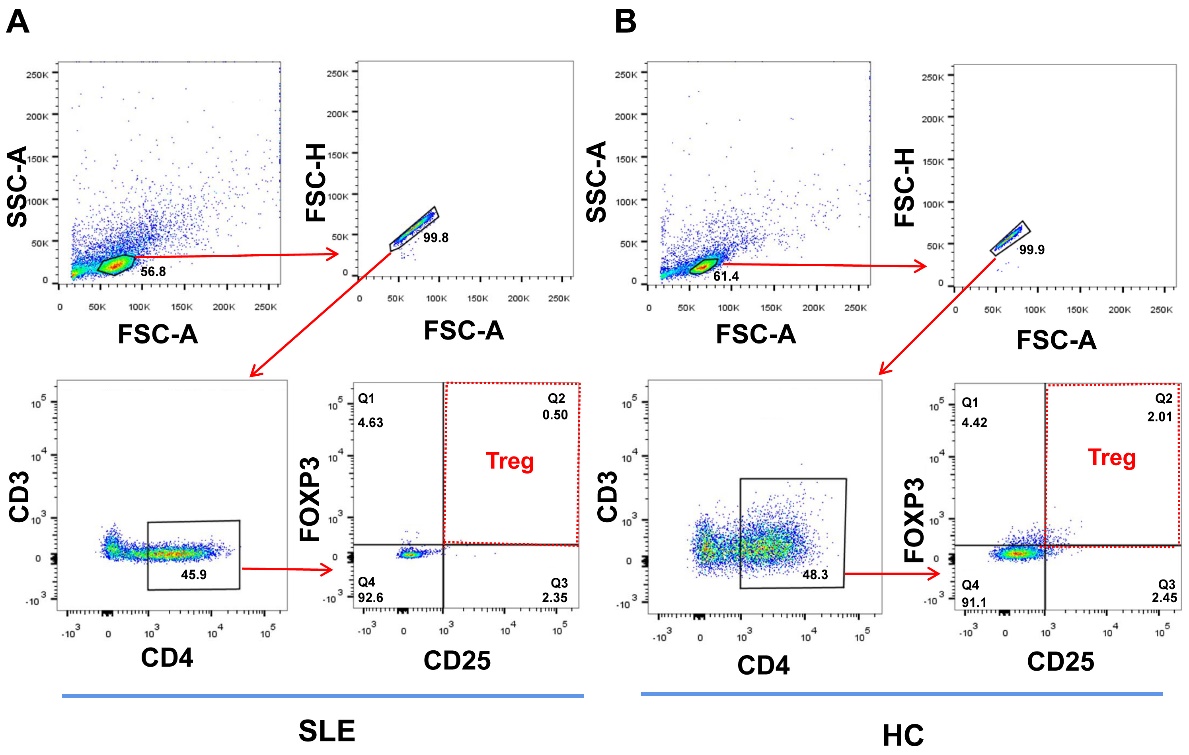
**

**Figure S2.** **Gating strategy for the detection of Treg cells.**

Figure represents the result of **(A)** the patient with SLE compared to **(B)** the healthy control. Lymphocyte population was gated from PBMCs according to forward scatter area (FSC-A) and side scatter area (SSC-A) characteristics. Discrimination of single cells was performed by plotting forward scatter height (FSC-H) against forward scatter area (FSC-A). Gating strategy to discriminate CD4^+^T cell populations was set using CD3-antibody and CD4-antibody. Treg cells (CD3^+^CD4^+^CD25^+^FOXP3^+^) were then gated from CD4^+^T cells.

**Additional file 5: Figure S3**


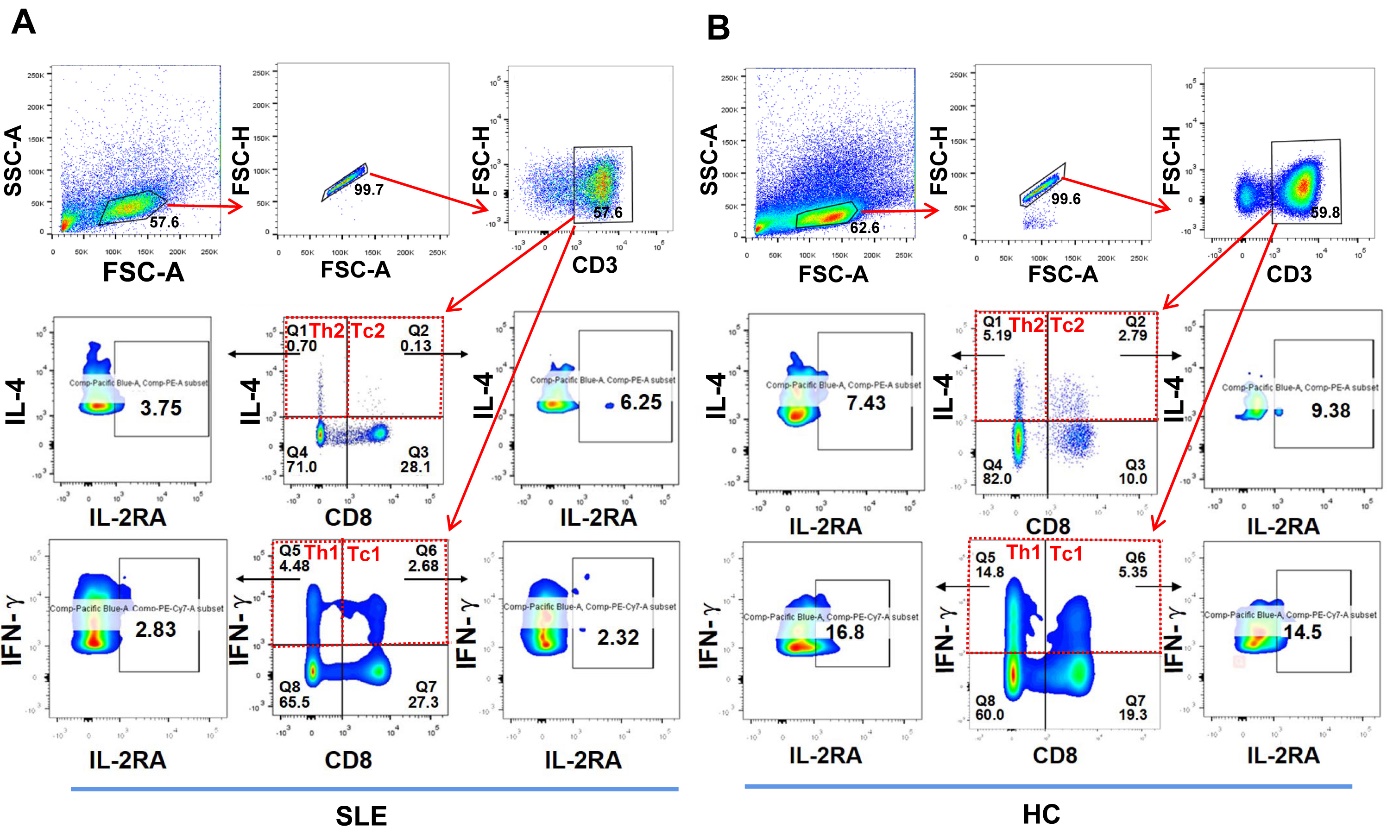


**Figure S3: Gating strategy for the detection of Th1, Th2, Tc1 and Tc2 cells.**

Representative examples of **(A)** the patient with SLE compared to **(B)** the healthy control. Lymphocyte population was gated from PBMCs according to forward scatter (FSC) and side scatter (SSC) characteristics. Discrimination of single cells was performed by plotting forward scatter height (FSC-H) against forward scatter area (FSC-A). Gating strategy to discriminate T cell populations was set using CD3-antibody. Th1 (CD3^+^CD8^-^IL-4^high^), Th2 (CD3^+^CD8^-^IFN-γ^high^), Tc1 (CD3^+^CD8^+^IFN-γ^high^) and Tc2 (CD3^+^CD8^+^IL-4^high^) cells were then gate from T cell population by using the indicated antibodies. IL2RA expression in Th1, Th2, Tc1 and Tc2 subpopulations from SLE patients and healthy controls was detected by staining with pacific blue labeled anti-human IL2RA-antibody.
